# Linguistic markers of deception in Arabic news headlines: A cross-corpus study of stylistic and numeric features

**DOI:** 10.1371/journal.pone.0356823

**Published:** 2026-08-26

**Authors:** Eman Salamah Albtoush, Keng Hoon Gan, Saif Addeen A. Alrababah

**Affiliations:** 1 School of Computer Sciences, Universiti Sains Malaysia, Gelugor, Malaysia; 2 Faculty of Information Technology, Mutah University, Karak, Jordan; 3 Faculty of Information Technology, Al alBayt University, Mafraq, Jordan; University of Kerbala, IRAQ

## Abstract

The rapid spread of misinformation in Arabic news headlines poses a growing challenge to digital media integrity, given the scarcity of Arabic-specific automated detection tools relative to English-centric systems. Headlines are brief and context-limited, yet their lexical and stylistic patterns encode strong cues of veracity or deception, making headline-only detection practically urgent and linguistically tractable. This study investigates automatic fake-news detection using Arabic headlines, leveraging five heterogeneous corpora and their unified combination. English sets were incorporated via neural machine translation with light post-normalization that preserves stylistic cues, yielding a heterogeneous cross-domain corpus. A systematic analysis of linguistic and stylistic indicators reveals stable asymmetries between fake and real headlines that recur across domains. We evaluate approaches from classical TF-IDF baselines to Arabic-specialized transformers, and propose a late-fusion strategy coupling transformer representations with discriminative engineered features. Transformers consistently outperform classical baselines, confirming that subword representations effectively capture semantic and stylistic regularities in short Arabic texts. Late fusion yields statistically significant improvements only on datasets with prominent numeric or temporal cues; on the unified corpus, McNemar’s exact test confirms that fusion gains are non-significant, indicating that subword encoders already internalize the surface-level cues captured by the engineered features. Even where accuracy differences are marginal, interpretable features enhance explainability.

## 1. Introduction

Arabic is a major world language, spoken by hundreds of millions of people. It is estimated to be the fifth most spoken language globally with around 422 million speakers [1], serving as the first language for Arab countries and holding significant cultural importance. Arabic’s morphology is notably rich: it uses a root-and-pattern system with on the order of 10,000 roots and more than 900 patterns [2].This templatic derivational structure yields a vast vocabulary of related word forms from each root. Additionally, Arabic exists in multiple varieties – Classical (Quranic) Arabic, Modern Standard Arabic (MSA), and numerous colloquial dialects – which differ phonologically, syntactically, and lexically. These factors combine to make Arabic highly inflectional and complex [3,4]. In Natural Language Processing (NLP) terms, such complexity poses major challenges: free word order, rich affixation, ambiguous diacritics (vowel markings), and dialect variation all complicate tasks like tokenization, parsing and semantic analysis. In particular, fake news detection in Arabic faces “unique challenges” such as this “complex grammar, diverse dialects, and the scarcity of annotated datasets” [5]. The rapid spread of false information (“fake news”) is a global concern, and Arabic social media and news outlets have been fertile ground for misleading headlines. Fake news has been called ’one of the deadliest weapons’ against societies [6], threatening the public mind, social stability, and even public health. Its spread is amplified by social networks, which enable sensational or misleading headlines to go viral. Studies note that user-shared headlines that provoke fear or outrage tend to propagate much faster on social media, especially in crisis situations [7]. For example, one widely circulated piece of fake news on social media falsely attributed to the news agency Reuters claimed that Saudi King Salman bin Abdulaziz Al Saud had died at the age of 89. The post, shared on the platform X, garnered over 283,000 views, 128 shares, and 1,837 likes before it was flagged as false by the fact-checking platform Sawab [8]. Another case, identified by Misbar as misleading, asserted that WhatsApp had introduced a new update to automatically create groups with the two contacts a user communicates with most frequently [9]. Similarly, FullFact also debunked a viral claim alleging that the World Health Organization (WHO) had declared the end of the COVID-19 pandemic. In reality, the WHO had only announced the termination of COVID-19 as a global health emergency, not the pandemic itself [10]. These examples highlight how misleading or sensational Arabic-language headlines—whether related to politics, technology, or health—can rapidly reach large audiences across social media. Because headlines are typically short and striking, many readers accept such claims without verification, reinforcing misinformation and shaping public opinion in ways that may significantly affect beliefs and behaviors.

News headlines – including those in Arabic – are typically very concise (often around 8–12 words on average [11], and are crafted to capture attention [12]. Arabic headlines, especially in Modern Standard Arabic, tend to omit vowels (diacritics) and rely heavily on context. The limited word count means each word carries weight, and stylistic conventions can serve as important cues. For example, headlines often include **question marks** such as: هل هذا الخبر صحيح؟ (Is this news true?), ماذا يحدث في المنطقة؟ (What is happening in the region?), من يقف وراء القرار المفاجئ؟ (Who is behind the sudden decision?), لماذا ارتفعت الأسعار؟ (Why did prices increase?), and أين اختفى المسؤول السابق؟ (Where did the former official disappear?). They may also employ **exclamation words** such as: !عاجل (Breaking!), !خطير (Dangerous!), !مفاجأة (Surprise!), !صادم (Shocking!), !لا يصدق (Unbelievable!), !بالفيديو (With video!), !انظر ماذا حدث (Look what happened!), !احذر (Beware!), and !كارثة (Disaster!). In addition, the inclusion of **numbers** or other stylistic markers can further strengthen the persuasive or deceptive tone of a headline. From an NLP perspective, headlines are short texts from which machine learning models can extract a variety of features. Possible features include word-level representations (such as TF-IDF weights or word embeddings), character n-grams, and statistical patterns (e.g., word frequencies). One study on Arabic headline classification noted that transformer models can capture “nuanced linguistic features relevant to fake news detection in Arabic” [12], implying that subtle semantic or contextual clues are present even in headlines. Other useful features may include morphological analyses (root/stem patterns, prefixes/suffixes). For instance, many Arabic words share the same root such as قتل (to kill), which generates forms like قاتل (killer), مقتول (killed), اقتتال (fighting), and مقتلة (massacre). Prefixes such as الـ (the) and بـ (with/by) or suffixes like ون (plural masculine, e.g., مجرمون – criminals) and (plural feminine, e.g., ضحايا – female victims) [13] can significantly alter meaning and grammatical role, offering valuable cues for fake news detection, Similarly, the root كتب (to write) yields كاتب (writer), مكتوب (written), كتاب (book), and مكتبة (library), illustrating how one root spans agent, patient, and place meanings. To make this root-and-pattern system concrete, Table 1 gives worked examples of how two triliteral roots generate several related word forms, together with common affixes that change grammatical role.

**Table 1:** Illustrative examples of Arabic root-and-pattern morphology and affixation, using the triliteral roots ق ت ل (q-t-l, “to kill”) and ك ت ب (k-t-b, “to write”).

Form	Translit.	Gloss	Morphological process
*Root* ق ت ل *(q-t-l)*			
قتل	qatala	“he killed”	Base verb (root realization)
قاتل	qatil	“killer”	Active participle
مقتول	maqtul	“killed (one)”	Passive participle
اقتتال	iqtital	“fighting”	Verbal noun (Form VIII)
مقتلة	maqtala	“massacre”	Noun of place / intensity
*Root* ك ت ب *(k-t-b)*			
كتب	kataba	“he wrote”	Base verb (root realization)
كاتب	katib	“writer”	Active participle
مكتوب	maktub	“written”	Passive participle
كتاب	kitab	“book”	Verbal noun / noun
مكتبة	maktaba	“library”	Noun of place
*Common affixes*			
الـ	al-	“the”	Definite article (prefix)
بـ	bi-	“with / by”	Preposition (proclitic prefix)
ـون	-un	masc. sound plural	Suffix, e.g., مجرمون (criminals)
ـات	-at	fem. sound plural	Suffix, e.g., معلمات (female teachers)

Linguistic features such as part-of-speech (POS) tags, syntactic structures, and lexical markers of sensationalism can provide strong signals for assessing the veracity of headlines. For instance, POS tags can reveal patterns such as frequent use of verbs in the imperative form, e.g., اكتشف الحقيقة الآن (“Discover the truth now”). Syntactic structures may include interrogatives that raise doubt or curiosity, such as هل هذا الخبر صحيح؟ (“Is this news true?”) or لماذا ارتفعت الأسعار؟ (“Why did prices increase?”). Lexical markers of sensationalism often appear as emotionally loaded adjectives or exaggerated terms, e.g., كارثة كبرى (“major disaster”) or صدمة مفاجئة (“shocking surprise”). These stylistic choices can exaggerate or distort reality, making them useful linguistic cues for fake news detection in Arabic headlines. Preprocessing steps such as removing stopwords, punctuation, and diacritics have often been applied in recent studies to normalize Arabic headlines [14]. However, these seemingly minor elements may themselves carry valuable signals for fake news detection. For example, the frequent use of stopwords like هذا (“this”) or تلك (“that”) in a headline may indicate vague reference and sensational framing. Punctuation can also be a strong cue, with repeated exclamation marks (!!!) or question marks (؟؟؟) often signaling exaggerated or misleading tone, as in هل سينهار الاقتصاد قريبا؟ً؟؟ (“Breaking: Will the economy collapse soon???”). Dialectal words, such as شو (“what”) or ليه (“why”), may also reveal attempts to target specific audiences and increase virality on social platforms. In this study, we therefore adopt a light pre-processing strategy, deliberately preserving stopwords, punctuation, dialectal tokens, and other subtle markers. By retaining these details, we aim to maximize the amount of linguistic information available and better capture the stylistic and rhetorical cues that make fake headlines distinct.

Detecting fake news in Arabic (headline) text has been approached through a spectrum of methods, from manual verification to advanced machine learning. Traditionally, fact-checking organizations (e.g., Misbar in Saudi Arabia, Fatabyyano, digital media literacy initiatives) examine viral claims using human expertise [5]. These services can identify false or misleading headlines through research and source tracing, but they rely on labor-intensive manual processes. Such fact-checkers, while thorough, cannot scale to the massive volume of online content [7]. In response, researchers have developed automated content-based approaches. The earliest method is to use classical machine learning (ML) on textual features: extracting handcrafted features (e.g., word counts, TF-IDF vectors, lexical and syntactic cues) and training classifiers like SVMs or random forests. In fact, the literature notes that Arabic fake-news systems “mainly” follow two strategies: one based on ML with manually engineered statistical features, and another on end-to-end deep learning. Content-based ML has the advantage of interpretability and relatively low data needs, but its performance is limited by the quality of feature engineering [15]. Human-driven fact-checking is the baseline approach. For example, projects like Fatabyyano and Misbar maintain databases of debunked claims and highlight fabrications in Arabic headlines. However, fact-checking is reactive and expert-driven: it does not automatically flag news but corrects misinformation post fact. Its strengths are high accuracy (experts provide context and judgement) and adaptability to any content, but the drawback is clear: it is slow, resource-intensive, and unsuited for real-time detection. In practice, automated systems seek to augment or predict fact-checker decisions using computational models [7]. Traditional ML approaches treat fake-news detection as a text classification task. They first convert a headline (and possibly article body) into numeric features. Common features include term frequencies (TF-IDF), stylometric patterns (e.g., punctuation counts, headline length), readability scores, sentiment polarity, and grammar cues. As Abbas et al. explain, ML methods “rely on manually produced statistical data extracted from the text” as features to distinguish real vs. fake [16]. For Arabic specifically, features often exploit morphology (e.g., using roots or stemmers) and lexical resources. These features feed models like SVM, logistic regression, or tree-based classifiers. Such ML models can be trained on labeled datasets (e.g., AFND [17], Arabic News Stance [18]). The advantages are that they are relatively simple and can incorporate expert knowledge, but their accuracy is often outmatched by deeper models. They may also struggle if the handcrafted features fail to capture subtle cues. Deep learning (DL) models use neural networks to automatically learn features from text. In recent Arabic fake-news work, common architectures include CNNs, RNNs/LSTMs, and hybrid ensembles. Deep models can operate end-to-end, ingesting raw (or embedded) words and producing a fake/real prediction without manual features. They can learn complex patterns in the data, but require larger annotated corpora and more compute. Hybrid models combine the strengths of different approaches. For example, Turki et al. propose WaraBERT, which fuses word-level tokenization with multiple AraBERT encoder variants in a BiLSTM classifier. Their WaraBERT system (a hybrid contextual feature approach) achieved higher accuracy than simple TF-IDF or vanilla AraBERT models. This suggests that leveraging both deep embeddings and specialized layers (e.g., LSTM) can improve detection. In general, deep/hybrid methods offer improved accuracy (by learning complex semantic features) but at the cost of needing large datasets and being less interpretable [14].The latest trend is to use transformer-based pre-trained language models (e.g., BERT) fine-tuned for fake-news classification. Arabic-specific models like AraBERT, Arabic BERT, and AraELECTRA have been applied to headline datasets. For instance, Albtoush et al. found that transformer models dramatically outperformed others: AraBERTv02 achieved 70.4% accuracy on a large Arabic headline dataset, and AraELECTRA 77% on another. These models’ advantage is their ability to capture contextual and semantic nuances from even a short text – exactly the “nuanced linguistic features” that matter in fake-news. The downside is computational cost: transformers require fine-tuning on GPUs and large memory, and they can overfit if data is scarce [12]. Even though headlines are short, a variety of features can be extracted for machine learning. For example, one can use lexical features such as word n-grams or TF-IDF vectors [16]. Morphological features are also useful: extracting roots or stems of Arabic words helps normalize inflections [19]. Named-entity recognition (NER), which identifies names of persons, locations, or organizations, can indicate whether a headline refers to specific individuals or events. For instance, the mention of a person like محمد صلاح (Mohamed Salah) or a place such as غزة (Gaza) may be used to draw immediate attention and lend credibility. Sentiment or affective features, such as overtly positive or negative adjectives, can also serve as strong signals of clickbait or propaganda—for example, headlines containing words like رائع (“wonderful”), مروع (“horrific”), or صادم (“shocking”). Similarly, syntactic features such as part-of-speech (POS) tag sequences or simple grammatical patterns can distinguish factual from deceptive headlines. For example, a declarative structure like الرئيس يعلن عن مبادرة جديدة (“The president announces a new initiative”) may reflect a factual tone, whereas an interrogative structure like هل ينهار النظام قريباً؟ (“Will the system collapse soon?”) could be indicative of sensational or speculative content [14]. Advanced models supplement such hand-crafted features with word embeddings or transformer-derived vectors, which implicitly encode meaning and context [12]. The ensemble of lexical, morphological, and learned embedding features provides the input for machine learning classifiers to detect fake Arabic headlines. Table 2 shows Summary of methods for detecting fake news in Arabic headlines: key strengths and limitations.

**Table 2:** Summary of methods for detecting fake news in Arabic: key strengths and limitations.

Method / Approach	Advantages	Limitations
Manual Fact-Checking	Very high accuracy and contextual understanding; uses expert judgment.	Labor-intensive, slow, and not scalable to large volumes; cannot automatically flag content in real time [5].
Classical Machine Learning	Simple models (SVM, logistic regression, decision trees) using handcrafted features (n-grams, TF–IDF, stylometric cues). Interpretable and efficient with limited data.	Requires manual feature engineering; may miss complex semantics; performance depends on feature richness [20].
Deep Learning (CNN/LSTM)	Learns hierarchical features directly from text; captures patterns across words; no manual feature design required.	Data-hungry; many parameters; prone to overfitting on small datasets; less interpretable [21].
Hybrid / Ensemble Models	Combine multiple techniques (e.g., embeddings + LSTM; multiple feature sets). Often improves accuracy.	More complex to design and tune; requires larger data and compute; integration overhead [22].
Transformer Models	Contextualized embeddings (e.g., AraBERT, AraELECTRA) capture nuanced semantics; state-of-the-art performance on Arabic text.	Resource-intensive (GPU/memory); large model size; risk of overfitting; fine-tuning can be slow [23].

To clarify the novelty of this study, our contribution goes beyond applying existing classifiers to a single Arabic fake-news dataset. First, we focus specifically on headline and only headlines Arabic fake news detection, extending our earlier line of work [12] on Arabic headline classification toward a broader multi corpus setting. Second, to address the scarcity of Arabic fake news resources, we enrich the evaluation space by translating two widely used English benchmark datasets, LIAR and WELFake, into Arabic via MT-based data augmentation, thereby enabling a controlled multi-source assessment of fake news detection in short Arabic text such as headlines. Third, we incorporate the multi dialectal VERA–ARAB dataset to examine how models trained on or exposed to Modern Standard Arabic and dialectal Arabic behave under domain and register variation, particularly when social-media language is mixed with Arabic news. Finally, we introduce a dataset-specific and unified feature-fusion framework in which the most informative engineered linguistic and stylistic cues are identified for each corpus, as well as for the merged corpus, and then fused with text representations. This allows us not only to compare linear, hybrid, and transformer models under a unified setup, but also to better understand which linguistic cues remain robust across native Arabic, translated Arabic, and dialect-rich settings.

## 2. Related work

The scarcity becomes even more pronounced when focusing on **headlines**, which are a primary entry point for many readers. Large scale analyses of online behavior reveal that over 75% of shared news links on Facebook were never clicked before resharing, underscoring the influence of headlines on information consumption [24]. Detecting fake news in Arabic poses unique challenges due to the language’s rich morphology, dialectal variation, and scarcity of annotated data [5]. Early Arabic FND work relied on hand-crafted linguistic features. For example, Himdi et al. (2022) extracted lexical and stylistic cues (e.g., word lists for sentiment, emotion, part-of-speech patterns) from Arabic news articles. Using these psycholinguistic and polarity features, a random forest model achieved about 78% accuracy. This study showed that combining lexical, emotional, and syntactic cues yields better performance than any single feature type. Similarly, satirical fake news in Arabic exhibits distinct linguistic markers: Saadany and Mohamed (2020) analyzed an Arabic satire news corpus (∼3,185 satirical vs 3,710 real articles) and found that lexico-grammatical features effectively discriminate satire from real news, allowing an ML classifier to reach up to 98.6% accuracy [25]. These works highlight that lexical diversity, readability and rhetorical style can reveal deception in Arabic news. In parallel, deep learning and contextual embedding approaches have become state-of-the-art. Bou Nassif et al. (2022) constructed a large diverse Arabic fake-news dataset and fine-tuned several pre-trained Arabic Transformer models. They report that advanced contextual embeddings (e.g., variants of AraBERT, QARiB) are very robust, with overall detection accuracy exceeding 98% [26]. Aboulola and Umer (2024) similarly combined contextual ELMo embeddings with a CNN–LSTM ensemble: their hybrid model on a large public Arabic news corpus (AFND, 606k articles) achieved 98.4% accuracy [7]. Umer et al. (2024) further showed that stacking ensembles of multiple embedding-based classifiers (bagging + boosting) can push performance even higher (almost 99% accuracy, with precision/recall 99%) [27]. These results, along with Al-Yahya et al. (2021) who found a large F1 improvement (from 0.83 to 0.95) when replacing GRU networks with transformer-based encoders [28]. underline that deep contextual models consistently outperform older feature-based models in Arabic FND. Researchers have also explored feature fusion techniques. Dahou et al. (2024) proposed an ensemble combining deep networks (Bi-GRU and Bi-LSTM) with linguistic features and NER cues, selected via reinforcement learning and swarm optimization. On public Arabic datasets (AFND and a Twitter fake-news corpus), their model achieved very high recall/F1 (0.98–0.99) and accuracy [29]. Rashed et al. (2024) built a unified Arabic fake-news corpus (AMFND) and trained an ensemble of fine-tuned transformer models. Using weighted voting, their ensemble reached an F1 of 0.98 (accuracy 0.98) on test data, significantly outperforming any single model [30]. These hybrid approaches show that combining language-level cues (sentiment, entity presence, readability) with semantic/contextual encodings improves robustness, albeit at cost of complexity and data requirements. Challenges remain: Arabic dialects, infrequent classes (some fake-news types), and limited labeled corpora. For example, the AFND dataset contains mostly Modern Standard Arabic (news domains), so models may not generalize to social media or dialectal Arabic. Many studies note that adding more data (e.g., from multiple sources or synthetic generation) and refining features (e.g., satire vs malicious intent) are important future steps. In sum, the literature shows that linguistic analysis (from simple counts to NER and stylometry) is a valuable complement to deep learning in Arabic FND [28].

### 2.1 Arabic fake news in headlines

Fake news often propagates via sensational headlines, so some studies focus specifically on headlines. Najadat et al. (2022) studied Arabic headline–article pairs in Middle East war news. They trained an LSTM and a CNN–LSTM ensemble to judge if a headline matches the article. The CNN–LSTM yielded about 70% accuracy in distinguishing true vs misleading headline-article pairs [31]. This relatively modest accuracy (compared to full-article detection) reflects the difficulty of verifying claims solely from headlines. Other work on headline-style deception includes clickbait detection: Bsoul et al. (2022) compiled a Jordanian Arabic headline dataset (∼3,000 news tweets) labeled as “clickbait” or not. Traditional ML models (Logistic Regression, SVM, etc.) on this data achieved a macro F1 of about 0.81 [32]. This indicates that superficial features of headlines (length, punctuation, sensational words) can signal deceptive marketing content. Few studies have targeted standalone Arabic headlines for fake-news classification beyond clickbait. Al-Btoush et al. (2025) recently evaluated classical ML vs deep models on Arabic headlines,they mention that transformer-based classifiers outperform LSTM or SVM on labeling headlines as fake or real [12]. However, comprehensive results are still emerging. Table 3 shows the Comprehensive summary of studies on Arabic fake news detection through linguistic features, from general text to headline-only settings, including methods (ML/DL/transformers), datasets, reported Accuracy/F1, key findings, and limitations.

**Table 3:** Comprehensive summary of studies on Arabic fake news detection through linguistic features (2020–2025), from general text to headline-only settings, including methods (ML/DL/transformers), datasets, reported Accuracy/F1, key findings, and limitations.

Year	Methods	Dataset	Accuracy / F1	Key Findings	Limitations
[28] 2021	Deep learning such as CNN, RNN vs Arabic transformer	Multi-corpus (four datasets 200k), health domain	QARiB on ArCOV19-Rumors (0.795), and AraBERT (0.958)	AraBERT v02 outperformed all compared models in terms of generalization	Single domain; limited feature analysis and huge class imbalance
[33] 2022	Textual analysis (lexicon lists for emotion, polarity, POS); ML (RF, SVM)	Arabic news articles (crowdsourced)	~78% (best with RF)	Linguistics features extracted were the most dominant cues used to detect deceptive text in fake news	Limited dataset (1098); formal news focus
[31] 2022	Deep learning (1D-CNN–LSTM) on headline–article pairs	War news (422 headlines, 3042 articles)	70% (CNN–LSTM)	CNN on LSTM improves over LSTM alone; headline verification is difficult	Small, domain-specific (war in Syria); content-only features
[26] 2022	Classifiers (8 Arabic Transformers)	Large mixed news corpus (translated from English (16k) and Arabic (10k))	GigaBert-base on translated data (91.2%), ARBERT (98.8%) on collected Arabic dataset	Arabic BERT variants are highly effective	Large data needed
[32] 2022	Traditional ML (LR, SVM, RF, NB) for clickbait headlines	Headlines (3k; 18% clickbait)	0.81 (macro F1)	Headline cues enable clickbait detection with solid F1	Single-country
[34] 2024	Text-CNN	(5k + translated English)	86.2% (Acc)	1D-CNN attains near-perfect	Generalization unclear; overfitting risk
[12] 2025	Machine learning, deep learning, and Arabic transformer models	AFND and ANS datasets	70.41% AraBERTv2, 77% AraELECTRA	These results highlight the robustness and effectiveness of transformer architectures in capturing nuanced linguistic features relevant to fake news detection in Arabic	Headline-only input limits context; ANS dataset is unbalanced; performance sensitive to preprocessing choices (e.g., stemming)
[35] 2026	Systematic literature review (PRISMA-guided synthesis) on the explainability of Arabic FND models	24 studies synthesized	Reviewed Transformer/hybrid models reach ≈0.95 benchmark accuracy	Predictive accuracy has advanced faster than explainability; proposes a unified framework centreing trust, accountability, and adoption	persistent gaps in dialect coverage, robustness to AI-generated misinformation, and evidence-based explainability

## 3. Preprocessing

**Fig 1:**
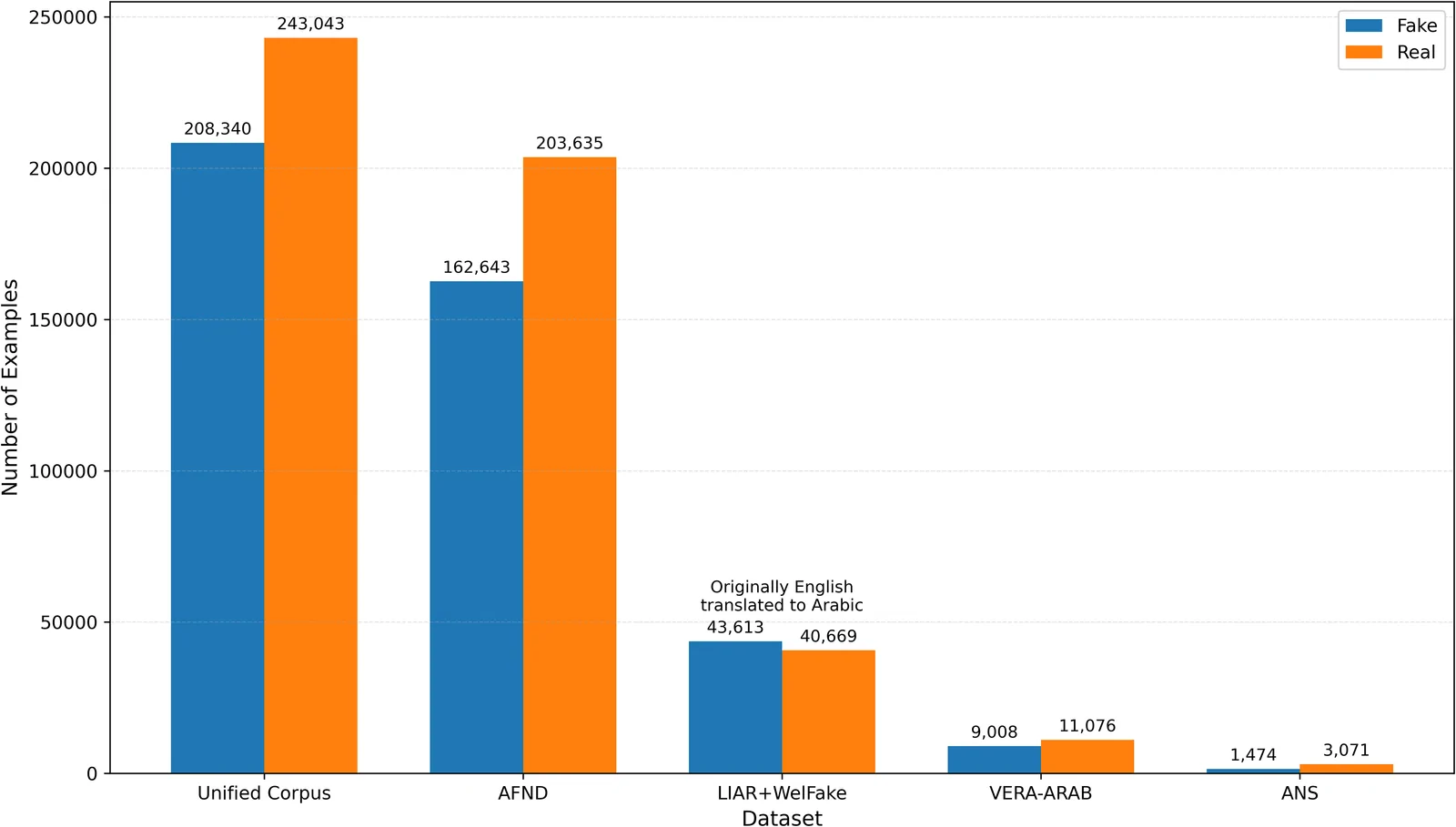
Distribution of Fake vs. Real headlines per dataset.

Our experiments draw on five publicly available datasets that together provide complementary coverage of domains, styles, and linguistic variety: **AFND** (large-scale, multi-country Modern Standard Arabic news with titles and full text) [17], **ANS** (headline/claim-focused with stance labels, well-suited to short-text veracity) [18], **VERA-ARAB** (multi-dialectal Arabic Twitter posts capturing informal and colloquial usage) [36], and the English **LIAR** [37] and **WeLFake** [38] corpora, which we translate to Arabic via machine translation to expand data diversity through data augmentation. AFND offers scale and topical breadth for supervised training; ANS stresses headline-only cues and stance-sensitive signals; VERA-ARAB injects dialectal variation and noisy social content to test robustness; LIAR and WeLFake contribute heterogeneous claim/news styles that strengthen generalization after translation. Note that AFND originally contains three credibility categories (credible, not credible, and undecided); in this study, we discard the undecided category and retain only the credible and not credible labels, reducing the task to a binary classification setting consistent with the other datasets. We apply preprocessing—normalizing obvious noise and duplicates while preserving potentially informative tokens (stopwords, punctuation, dialectal forms, numerals) to retain stylistic cues critical for headline-level fake news detection. This merged, multi-source setup is designed to reduce overfitting to any single domain, improve robustness to domain/dialect shift, and better evaluate models’ sensitivity to nuanced linguistic features. (Fig 1) shows the Distribution of Fake vs. Real Headlines per Dataset, and Table 4 summarizes each dataset’s characteristics used in this study.

**Table 4:** Comparison of AFND, ANS, LIAR, WeLFake, and VERA-ARAB datasets.

Ref	Year	Size	Label types	Domain
**AFND**	2022	606,912 articles	3-class (credible / not credible / undecided)	Arabic news (134 public sites, 19 countries)
**ANS**	2020	4,547 claims	2-class fake/real + stance (agree/disagree/other)	Arabic news headlines
**LIAR**	2017	12,800 statements	6-level truthfulness (Pants-on-fire, False, ..., True)	PolitiFact fact-checked statements
**WeLFake**	2021	~72,000 articles	2-class fake/real	News articles (mixed sources)
**VERA-ARAB**	2024	~20,000 tweets	2-class fake/real	Arabic Twitter (multi-dialectal)

### 3.1 ANS: Preprocessing, corpus statistics, and feature analysis

We apply an Arabic-aware normalization pipeline that mirrors our code: (i) replace surface artifacts with placeholders (<URL > , < EMAIL > , user mentions, hashtags; also collapse %/money, dates, and times to <PCT > / < MONEY > / < DATE > / < TIME>); (ii) unify Arabic digits to Latin; (iii) remove diacritics and *tatwīl*; (iv) normalize letter variants (و/ي→ؤ/ئ, ي→ى, ه→ة, ا→أ/إ/آ/ٱ); (v) collapse elongations; and (vi) tidy whitespace. We then drop empty, ultra-short (2 tokens) and duplicate texts while preserving row-wise alignment between the cleaned text matrix and engineered features. We obtain 4,545 unique claims: fake = 1,474, real = 3,071, Mutual information ranking highlights length/density and a few style/noise indicators as most predictive. Table 5 shows the engineered feature set used in ANS experiments. In the ANS corpus of short claims, several simple yet reliable linguistic cues distinguish real from fake content. Because the claims are compact, length and density measures—token and character counts as well as average token length—serve as stable discriminators. Logical markers such as negation and modal verbs are frequent and informative about authors’ degree of commitment versus speculation; a light future-tense signal offers additional leverage. Although numeric and temporal anchors (numbers, four-digit years, dates) are comparatively sparse, their presence typically aligns with verifiable statements and thus contributes disproportionately to identifying real claims. By contrast, sensational style indicators—trigger words, repeated letters or words, and emphatic punctuation—are rare overall but exhibit high precision when observed, providing complementary evidence for fake content. Finally, web or social artifacts (e.g., hashtags, ALL-CAPS bursts, explicit clock times) are largely absent and contribute little signal. These observations motivate a tiered modeling strategy: strong TF–IDF baselines over word and character boundary n-grams; hybrid models that append a compact, high-yield set of engineered features (length/density, negation/modality/tense, and numeric/temporal grounding) to the TF–IDF space; and transformer fine-tuning (AraELECTRA, MARBERT) optionally augmented by a normalized projection of the same top-K features.

**Table 5:** Engineered feature set used in ANS experiments.

Category	Features
Length / density	Character length, Token count, Average token length, Lexical diversity (type–token ratio)
Numeric / temporal grounding	Count of numbers, Has four-digit year, Count of dates, Count of money expressions, Count of percent expressions, Count of time expressions
Logical style	Count of negations, Count of modals, Count of hedging terms
Tense flags	Past tense flag, Present tense flag, Future tense flag
Sensational cues	Has question mark, Has exclamation mark, Multi-punctuation runs, Ellipsis present, Quote characters count, Has sensational trigger, Count of sensational triggers
Surface noise	Letter repetition flag, Word repetition flag, Typo ratio
Web / social signals (weak)	Number of URLs, Number of user mentions, Number of hashtags
Cross-script / CAPS heuristics	Number of English tokens, Has ALL-CAPS word
Entity / sentiment proxies	Pseudo named-entity count, Sentiment score (positive–negative per token)

This progression couples lexical evidence with factual and structural cues while avoiding overreliance on rare, dataset-specific artifacts. Table 6 illustrate ANS Per–class means for engineered features.

**Table 6:** ANS. per–class means for engineered features (sorted by |real−fake|, highest first). Top–10 rows are bold.

Feature	Fake mean	Real mean
**Character length**	**43.907734**	**50.602410**
**Token count**	**7.402985**	**8.452296**
**Negation count**	**0.881275**	**0.992511**
**Number count**	**0.074627**	**0.126994**
**Future tense flag**	**0.167571**	**0.195050**
**Question mark flag**	**0.012212**	**0.035819**
**Average token length**	**5.071066**	**5.085101**
**Modal verb count**	**0.034600**	**0.044285**
Four-digit year flag	0.011533	0.014002
Dialectal term count	0.008820	0.013676
Past tense flag	0.008141	0.011071
Hyperbole count	0.008141	0.010420
Date mention count	0.004749	0.009118
Hedging term count	0.004749	0.009118
Named-entity proxy count	0.004749	0.009118
Present tense flag	0.010176	0.006187
Negative emotion word count	0.001357	0.005210
Sensational trigger flag	0.002035	0.004884
Sensational trigger count	0.002035	0.004884
Repeated words flag	0.004749	0.003256
Money mention count	0.003392	0.003256
Positive emotion word count	0.002035	0.002279
Percent mention count	0.002035	0.001628
Repeated letters flag	0.002035	0.000977
Exclamation mark flag	0.001357	0.000977
English token count	0.000678	0.000651
ALL-CAPS word flag	0.000678	0.000651
Quotation mark count	0.000000	0.000651
Ellipsis flag	0.000000	0.000651
Multi-punctuation run flag	0.000000	0.000651
First-person pronoun flag	0.000000	0.000651
Multiple!/? flag	0.000000	0.000326
Has quotes flag	0.000000	0.000326
Simple sentiment score	0.000106	−0.000456

### 3.2 English datasets: LIAR and WELFake

**Fig 2:**
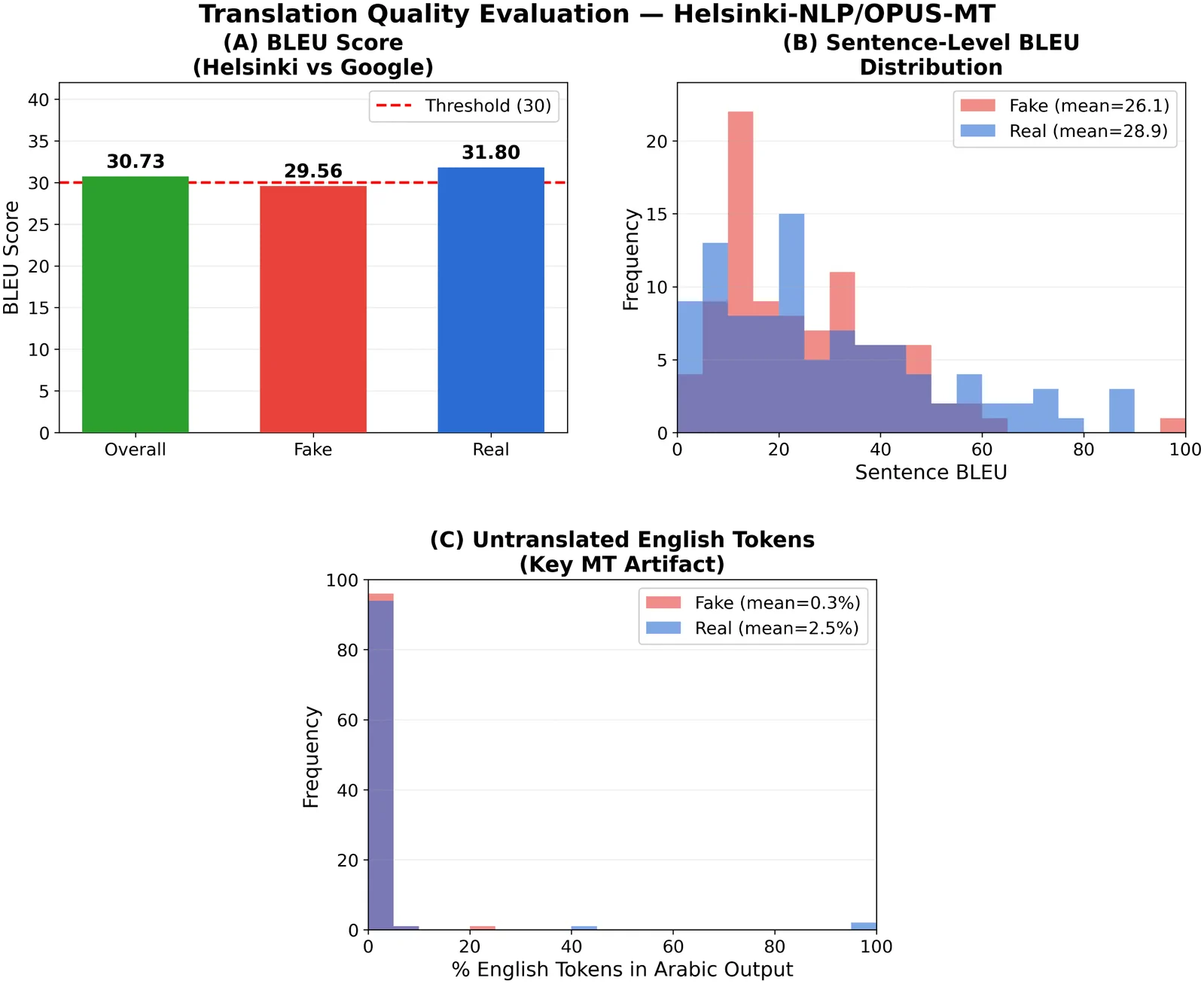
Translation quality evaluation of Helsinki-NLP/OPUS-MT on a stratified sample of 200 headlines. Feature comparison of fake versus real headlines in the Helsinki translation output. (A) Corpus-level BLEU scores against Google Translate as pseudo-reference; (B) sentence-level BLEU distribution per class; (C) proportion of untranslated English tokens in the Arabic output.

We build an Arabic headline corpus from the English LIAR and WELFake datasets by translating headline fields with **Helsinki-NLP/opus-mt-en-ar** (HELSINKI_MT) https://huggingface.co/Helsinki-NLP/opus-mt-en-ar, a Marian-based Transformer trained on OPUS. English titles are batched and translated on GPU; the resulting CSVs retain all original columns plus a HELSINKI_MT column with the Arabic output. To verify translation fidelity, we randomly sampled 200 headlines (100 fake, 100 real) from the merged corpus and evaluated HELSINKI_MT against Google Translate as a pseudo-reference using corpus BLEU [39] via sacreBLEU. The system achieved an overall BLEU score of 30.73 (fake: 29.56; real: 31.80), with sentence-level means of 26.1 and 28.9 respectively, consistent with published benchmarks for neural MT on Arabic news text. The narrow inter-class gap (2.24 points) indicates that translation quality does not systematically favour either class. Feature analysis of the Arabic output further identifies three categories of signals: MT artifacts (residual English tokens: 0.3% fake vs. 2.5% real; untranslated ALL-CAPS words), genuine stylistic signals (punctuation counts, character length), and mixed/ambiguous features (quotation marks, ellipsis) that may reflect both source style and MT formatting conventions. (Fig 2) summarises the BLEU evaluation and key MT artifact distributions; findings derived from translated data should be interpreted with the caveat that MT-induced patterns may partially inflate observed feature asymmetries, particularly for social-media artifacts such as hashtags and URLs.

Post-translation, we apply a loss-conscious, Arabic-aware normalization designed to preserve stylistic cues important for fake–news detection. We (i) replace volatile entities with typed placeholders and count them (<URL > , < EMAIL > , < PCT > , < MONEY > , < DATE > , < TIME>); (ii) unify Arabic/Latin digits; (iii) remove diacritics and tatwīl; (iv) normalize letter variants (أ/إ/آ/ٱ→ا, ة→ه, ى/ي→ي, ؤ→و, ئ→ي); (v) collapse excessive elongations; and (vi) standardize whitespace. Crucially, we preserve punctuation (" "؟! ...) and do not globally remove stopwords so that negation, modality, hedging, and teaser markers remain accessible. From the resulting text, we derive linguistic characteristics (counts / flags for punctuation, quotations, numbers, English tokens, hashtags, dates/times, negations, modals, hedges) and length measures. Class-wise feature means reveal a clear stylistic signature: fake headlines exhibit higher rates of hashtags, ellipses, multi-punctuation runs, exclamation/question marks, quotation usage, leftover English tokens (and ALL-CAPS), and numerical content (percentages, general numbers, years). They are also longer in characters/tokens, yet have slightly shorter average token length. Real headlines show relatively more explicit dates/times and mild increases in hedging. These patterns suggest deception-oriented or clickbait framing relies on sensational punctuation, teaser devices, social markers, and quantified claims, whereas real headlines more often convey temporal specificity and cautious phrasing. Table 7 reports corpus statistics following HELSINKI_MT translation and the above preprocessing.

**Table 7:** WELFake + LIAR (HELSINKI_MT) Corpus statistics after translation and preprocessing.

Item	Value	Notes
Total rows	84,282	merged LIAR + WELFake
Empty texts	16	removed
Duplicate texts	9,464	removed
Very short headlines (≤2 tokens)	707	removed
Label: fake	43,613	51.75%
Label: real	40,669	48.25%
Raw characters (min / max)	0 / 2,910	
Raw characters (mean / median)	76.07 / 70	
Raw tokens (min / max)	0 / 510	
Raw tokens (mean / median)	12.74 / 12	
Stopword tokens (sum)	107,100	fallback list
Content tokens (sum)	921,843	
Vocabulary size (excl. stopwords)	91,683	

Table 8 shows WELFake + LIAR (Helsinki-MT). Per–class means for engineered features. with a Top–10 rows (by absolute fake–real mean difference).

**Table 8:** WELFake + LIAR (Helsinki-MT). Per–class means for engineered features. Top–10 rows (by absolute fake–real mean difference) are shown first in bold.

Feature	Fake mean	Real mean
**Character length**	**76.236994**	**70.244093**
**Token count**	**12.858804**	**11.492660**
**Quotation mark count**	**0.479398**	**0.234306**
**English token count**	**0.252264**	**0.119428**
**Average token length**	**4.958458**	**5.077279**
**Has quotes flag**	**0.197556**	**0.102412**
**Number count**	**0.251026**	**0.168433**
**Multi–punctuation run flag**	**0.064797**	**0.003369**
Has exclamation mark	0.043152	0.001918
Question mark flag	0.053608	0.019868
Ellipsis flag	0.064316	0.003246
Multiple!/? flag	0.007567	0.000639
Symbol run count	0.001422	0.000393
ALL–CAPS word flag	0.045995	0.021638
Four-digit year flag	0.030541	0.022917
Date mention count	0.017128	0.022056
Negation count	1.657831	1.648184
Modal verb count	0.125719	0.103027
Hedging term count	0.017793	0.022376
Sensational trigger flag	0.004127	0.003270
Sensational trigger count	0.004196	0.003319
Number of percent expressions	0.009103	0.004795
Number of money expressions	0.001628	0.001033
Number of mentions	0.000390	0.000172
Number of hashtags	0.006741	0.000197
Number of times	0.000092	0.001328
Number of URLs	0.000252	0.000000

### 3.3 VERA-ARAB: Preprocessing, corpus statistics, and feature analysis

We apply the same Arabic-aware normalization used elsewhere in our pipeline. VERA-ARAB comprises 20,084 tweets; labels are 11,076 fake vs. 9,008 true. Domains are manually assigned across seven classes (religion, natural disaster, public security, armed conflict, public news, politics, sports) and are nearly balanced by fake/true; the largest shares are sports (~25%, 4,962 tweets; 2,586 fake) and politics (~20%, 2,366 fake). Claims were sourced from Misbar between Aug 8, 2022 and Sep 3, 2023.

Consistent with our headline-only goal, short tweet texts carry strong veracity signal: the original study reports promising results particularly with textual features, and positions VERA-ARAB as a balanced, multi-dialect benchmark for Arabic fake-news detection on social media. In our experiments, we prioritize lexical–stylistic indicators (length, density, character/word-level patterns, limited normalization) known to be effective for short texts; user/network features are excluded to prevent confounds.

Table 9 highlights robust separations between classes. Length and lexical richness are lower for Fake (fewer Unique words, smaller Token count, lower Guiraud’s R, and shorter Character length), suggesting more templated phrasing. Compression ratio is higher for Fake, indicating greater redundancy at the character level. Character composition also shifts: Fake shows a higher share of Latin letters % and slightly lower Arabic letters %, consistent with cross-lingual markers (e.g., brand names, handles, or Latin-script artifacts). Discourse cues diverge: Fake carries more Hashtags and marginally more URLs, while Real uses more Punctuation and notably more Emoji count. Finally, Fake exhibits larger Median word length yet fewer overall tokens and spaces, reinforcing the picture of concise but stylized constructions. These trends motivate our fusion design: a compact bundle of style/marker features augmenting MARBERT captures information orthogonal to pure semantics, improving headline-only detection without relying on user or network metadata.

**Table 9:** VERA_ARAB_feature_topN.

Feature	Fake mean	Real mean
**Unique words**	**17.906332**	**21.380820**
**Token count**	**19.373793**	**23.219716**
**Space count**	**18.373793**	**22.219716**
**Guiraud’s R**	**3.954171**	**4.296153**
**Character length**	**116.111097**	**135.708694**
**Compression ratio**	**0.762689**	**0.725555**
**Latin letters %**	**0.114698**	**0.091540**
**Emoji count**	**0.391348**	**0.570251**
**Median word length**	**4.977994**	**4.816852**
**Punctuation count**	**2.922257**	**3.536156**
Arabic letters %	0.652896	0.679434
Average word length	5.093842	4.971642
URLs	1.035737	0.960033
Hashtags	1.186583	0.979707
Spaces %	0.156619	0.160962

Class-wise means for engineered features (English, short names) on the VERA-ARAB dataset. The first ten rows—those with the largest absolute Fake–Real mean gaps—are shown in **bold**. Flag features are binary indicators (1 = present, 0 = absent); their means represent the proportion of headlines containing that feature.

### 3.4 AFND: Preprocessing, corpus statistics, and feature analysis

We merge the AFND real/fake files and apply the same Arabic-aware cleaning used for VERA–ARAB (placeholder URLs/mentions/hashtags, digit unification, removal of diacritics and tatwīl, letter normalization, collapsing of elongations, and deduplication of short or empty texts). Among the lexical richness features, we use Yule’s characteristic constant K as a measure of lexical repetition in a headline. For a given headline, let N denote the total number of word tokens, that is, the total number of words appearing in the headline after tokenization the words. Let $f_{i}$ denote the number of distinct word types that occur exactly *i* times in that same headline. For example, if a headline contains one word repeated twice and three other words appearing once each, then *f*_2_ = 1 and *f*_1_ = 3. Yule’s *K* is defined in Eq. 1 as:


$$\begin{array}{c} K=10^{4}\times \frac{\sum_{i\geq 1}i^{2}f_{i}-N}{N^{2}}. \end{array}$$ (1)

In this formulation, the term $\sum_{i\geq 1}i^{2}f_{i}$ captures how strongly words are repeated within the headline, because repeated words contribute more heavily through the squared frequency term *i*^2^. The subtraction of *N* and normalization by *N*^2^ make the measure comparable across headlines of different lengths. Importantly, the direction of this measure is inverse to many common diversity indices: higher values of *K* indicate greater lexical repetition and therefore lower lexical diversity, whereas lower values indicate a more varied vocabulary with less repetition. Class-wise feature means and correlations show clear Fake–Real stylistic gaps: (i) Fake headlines are slightly longer in characters/tokens but use shorter words on average; (ii) Fake is more repetitive lexically, exhibiting higher values of Yule’s characteristic constant (*K*), which quantifies lexical diversity by measuring the degree of word repetition—higher values indicate lower variety and greater lexical redundancy; (iii) character composition shifts toward more Latin letters and slightly more punctuation in Fake, while Arabic-letter share is marginally higher in Real; (iv) Fake carries more discourse or attention markers (hashtags, exclamation/question flags, modestly higher negation/modal terms); and (v) information-theoretic cues (compression ratio, character entropy) are slightly higher in Fake, hinting at redundant micro-patterns. These consistent signals (length/composition, lexical regularity, and simple discourse markers) complement semantic encoders and justify a hybrid fusion of compact stylistic features with transformer representations for headline-only detection. Table 10 highlights robust class separations.

**Table 10:** AFND_feature_means (sorted by |Fake−Real| descending).

Feature	Fake mean	Real mean
**Yule’s characteristic constant K**	**11.214656**	**8.275082**
**Character length**	**60.632004**	**59.856749**
**Space count**	**9.148940**	**8.897906**
**Token count**	**10.148940**	**9.897906**
**Punctuation count**	**1.394059**	**1.169720**
**Unique words**	**10.058355**	**9.841962**
**Average word length**	**5.117690**	**5.184004**
**Median word length**	**5.074510**	**5.126005**
**Word length std. dev.**	**1.841664**	**1.804009**
**Guiraud’s R**	**3.126826**	**3.100007**
Has quotes (flag)	0.116556	0.129300
Arabic letters %	0.811639	0.819674
Character entropy	3.915748	3.907986
Negation count	0.062487	0.055192
Digit count	0.603561	0.596995
Has exclamation (flag)	0.018076	0.011972
Punctuation %	0.022483	0.019456
Latin letters %	0.004836	0.001992
Compression ratio	0.822259	0.819529
Hapax ratio	0.992683	0.994972
Type–token ratio	0.993274	0.995384
Unigram diversity	0.993274	0.995384
Spaces %	0.150477	0.148525
Date mentions	0.004937	0.003000
Simpson’s D	0.891188	0.889593
Modal count	0.010274	0.008923
Money expressions	0.001316	0.002293
Has question mark (flag)	0.030047	0.029219
Hashtags	0.001125	0.000300
Herdan’s C	0.997175	0.997998
Percent expressions	0.007452	0.007901
Maas a2	0.001139	0.000834
Bigram diversity	0.999730	0.999889
Digits %	0.010622	0.010487
Time mentions	0.000184	0.000069
Reporting verb count	0.005577	0.005662
Starts with marker (flag)	0.000184	0.000103
Trigram diversity	0.999919	0.999992
Emoji count	0.000406	0.000457
User mentions	0.000049	0.000000
URLs	0.000012	0.000005

Per–class means for engineered features on the AFND fake/real headlines dataset. Top–10 rows (largest absolute class gaps) are in **bold**. Flag features are binary indicators (1 = present, 0 = absent); their means represent the proportion of headlines containing that feature.

### 3.5 Unified corpus construction: Preprocessing, corpus statistics, and feature analysis

To strengthen generalization and reduce bias in the data set, we consolidated five news headline sources into a single Arabic corpus by merging each item’s text field with its binary label. Specifically, ANS and AFND were used as is; LIAR and WELFake (originally English) were translated into Arabic before merging; and VERAARAB was added in its native Arabic form. We applied the same light, consistent pipeline across all sources (tokenization‐friendly cleanup and field normalization) while deliberately preserving surface cues (e.g., punctuation, numerals, quotation marks, code-switching) so that linguistic signatures that help distinguish fake from real content remain available to the models. The resulting unified dataset contains 451,383 headlines (Real: 243,043, Fake: 208,340). We then calculated a rich set of engineered features and inspected the per-class means; Table 11 reports the top 20 features by the absolute false-real mean gap for the unified corpus (the first ten rows are in bold).

**Table 11:** Class-wise means for engineered features on the unified ANS+AFND+LIAR+WELFake+VERA-ARAB corpus. The first ten rows — those with the largest absolute Fake–Real mean gaps — are shown in bold.

Feature	Fake mean	Real mean
**Yule’s K**	**15.326786**	**11.987348**
**Character length**	**63.287602**	**64.168092**
**Unique words**	**10.488293**	**10.560378**
**Token count**	**10.634161**	**10.701965**
**Space count**	**9.634161**	**9.701965**
**Punctuation count**	**1.348309**	**1.296607**
**Average word length**	**5.099633**	**5.149726**
**Word length std. dev.**	**1.870439**	**1.827638**
**Median word length**	**5.059682**	**5.083731**
**Has quotes (flag)**	**0.115012**	**0.137228**
Digit count	0.554050	0.571208
Has exclamation (flag)	0.014522	0.021106
Has question mark (flag)	0.028775	0.033516
Arabic letters %	0.809500	0.814126
Guiraud’s R	3.178777	3.182409
Latin letters %	0.009527	0.006777
Reporting verb count	0.009418	0.006941
Hapax ratio	0.989637	0.991587
Compression ratio	0.812902	0.811041
Unigram diversity	0.990361	0.992002

Class-wise means on the unified ANS+AFND+LIAR+WELFake+VERAARAB corpus; the first ten rows–those with the largest absolute Fake–Real mean gaps are shown in **bold**. Flag features are binary indicators (1 = present, 0 = absent); their means represent the proportion of headlines containing that feature.

**Fig 3:**
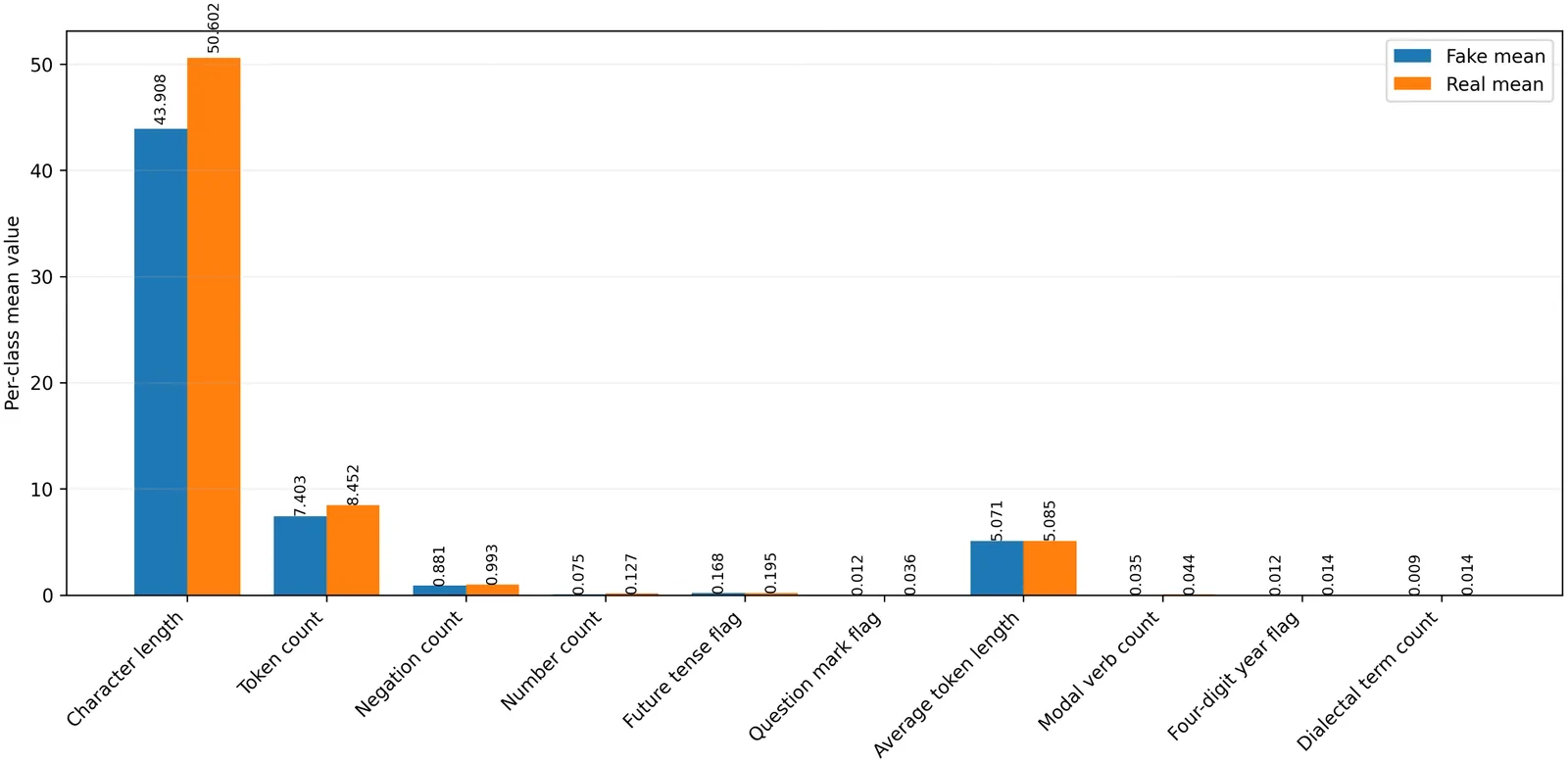
ANS — Top-10 engineered features (per-class means: Fake vs Real).

**Fig 4:**
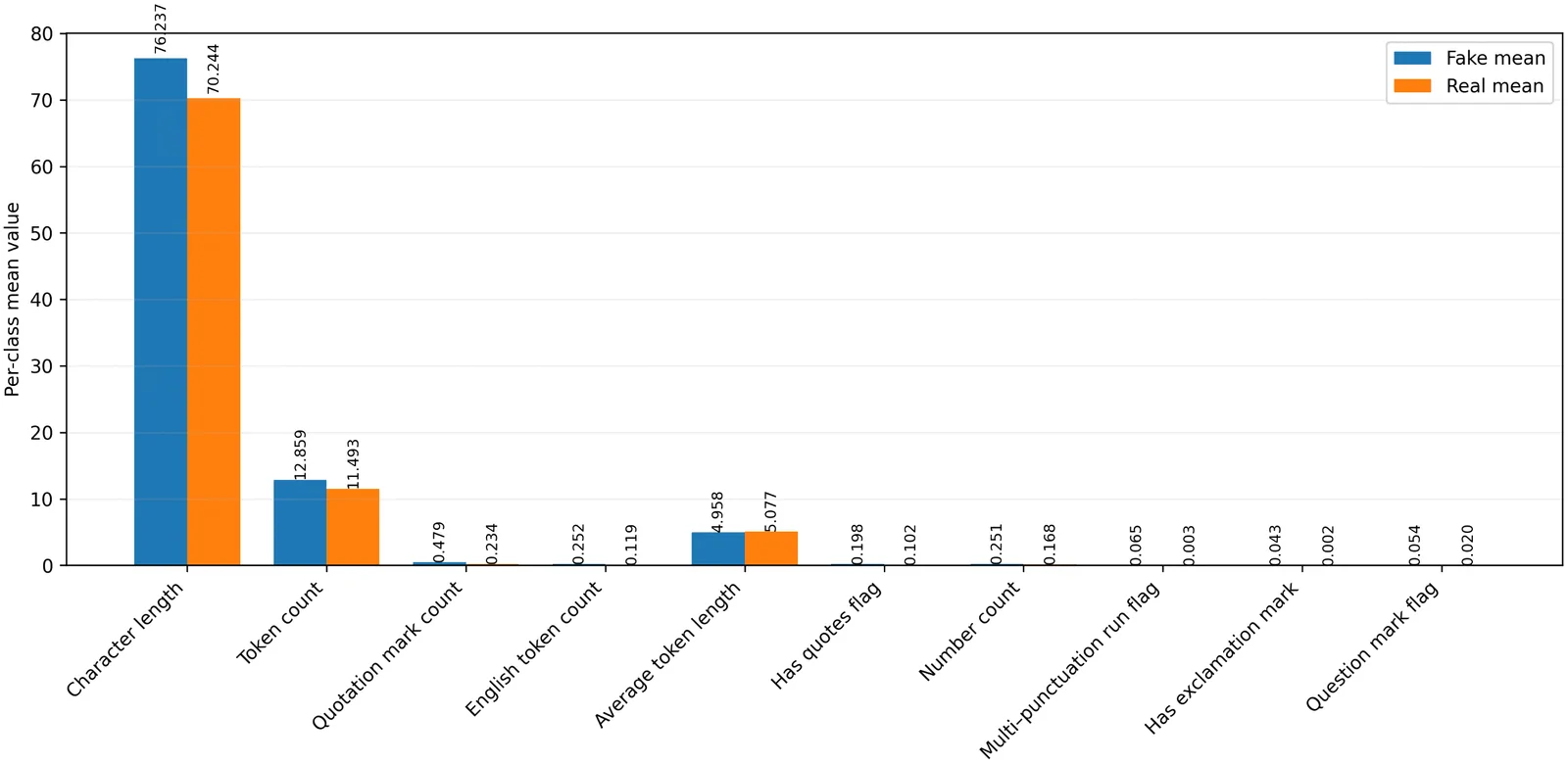
WELFake + LIAR (Helsinki–MT) — Top-10 engineered features (per-class means: Fake vs Real).

**Fig 5:**
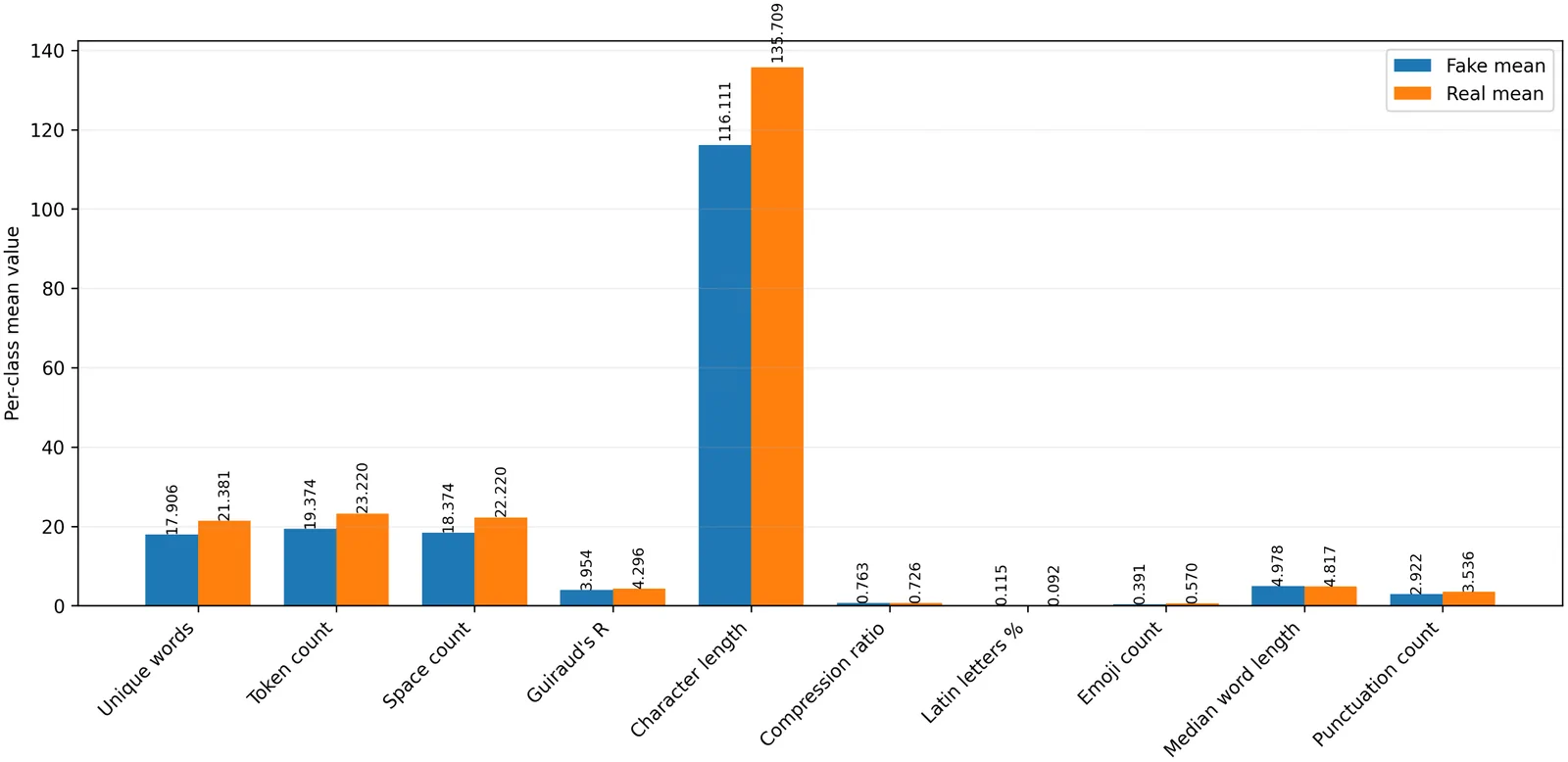
VERA–ARAB — Top-10 engineered features (per-class means: Fake vs Real).

**Fig 6:**
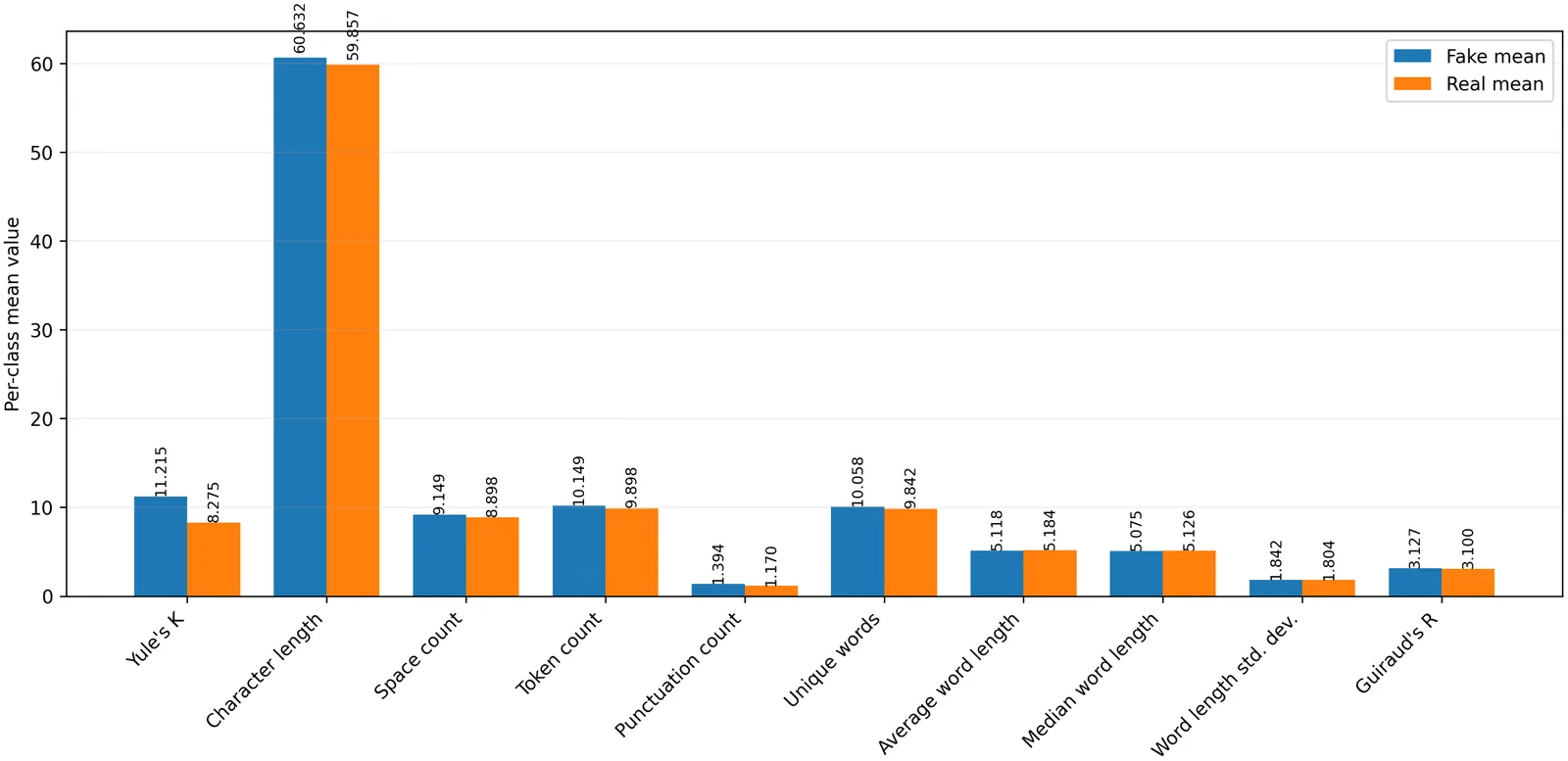
AFND — Top-10 engineered features (per-class means: Fake vs Real).

**Fig 7:**
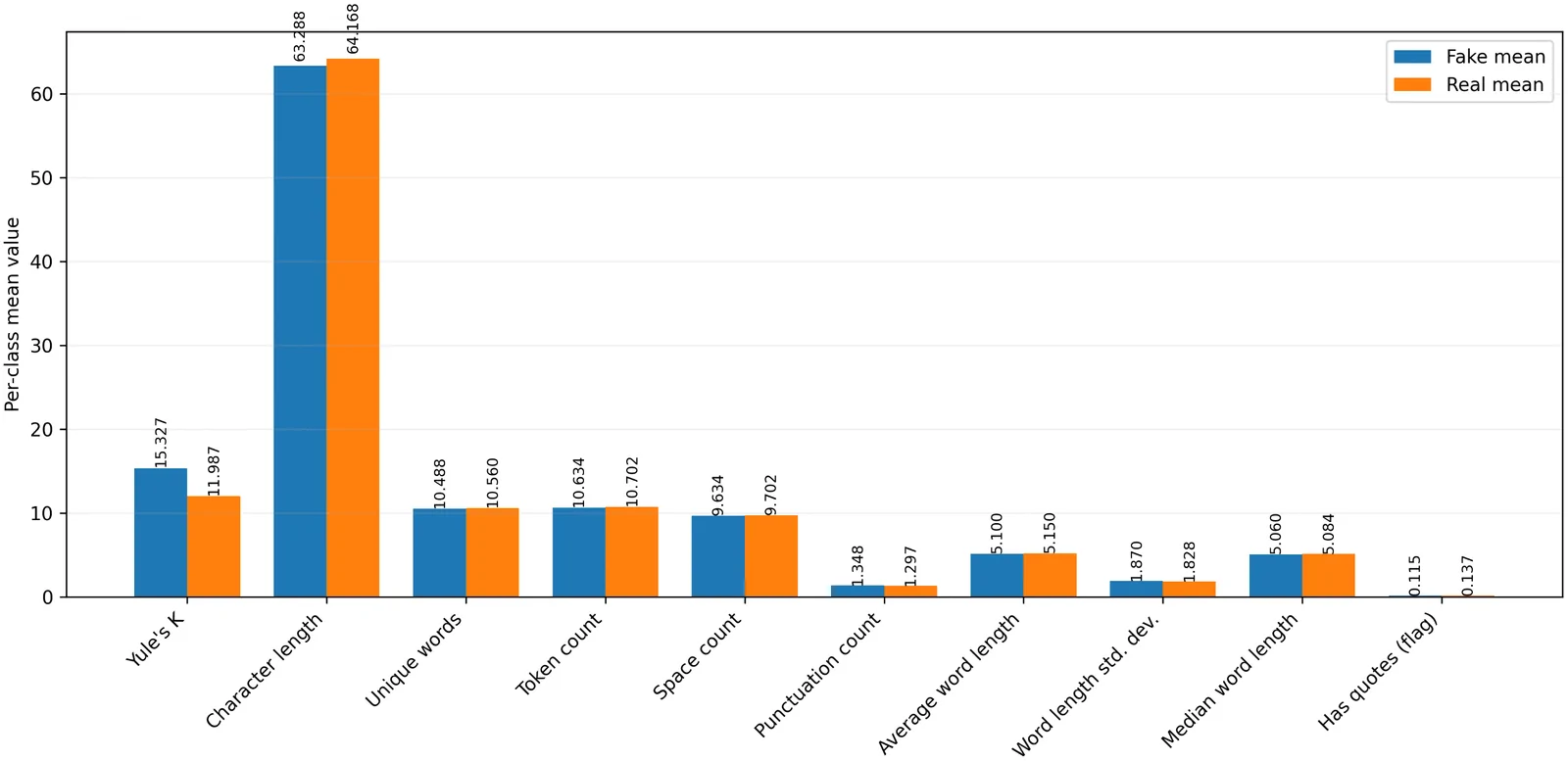
Unified corpus (ANS+AFND+LIAR+WELFake+VERA-ARAB) — Top-10 engineered features (per-class means: Fake vs Real).

Figs 3–7 illustrate the ten most discriminative linguistic and structural features across all evaluated datasets, contrasting Fake and Real class means. Because these engineered indicators vary in scale (counts, ratios, binary flags), bar heights are intended for within-feature comparison rather than absolute magnitude. Overall, the figures reveal that fake headlines tend to exhibit shorter character lengths, fewer tokens, and lower lexical richness, whereas real headlines are generally longer and syntactically denser.

**Fig 8:**
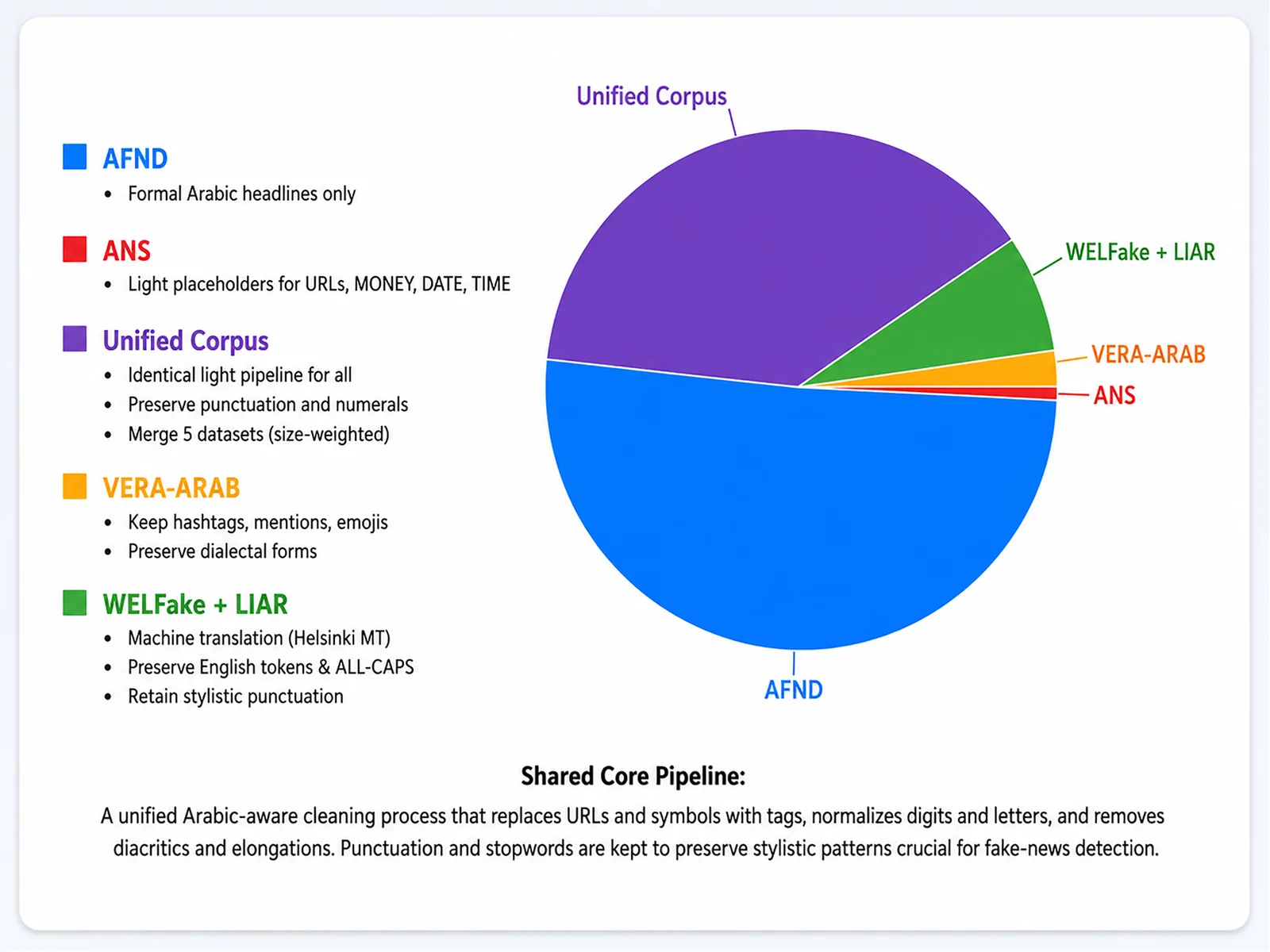
The Arabic fake-news datasets — Preprocessing pipeline summary.

Features such as punctuation frequency, negation usage, and the presence of numerals or quotation marks also show notable divergence, reflecting stylistic and rhetorical patterns that writers employ when crafting deceptive versus factual headlines. For the Unified corpus—an aggregated dataset combining ANS, AFND, WELFake, LIAR, and VERA–ARAB—the top ten distinguishing features confirm the robustness of these stylistic cues across domains. The most influential variables include Yule’s characteristic constant (K), character length, unique word count, and token count, each capturing distinct aspects of lexical diversity and content density. Fake headlines typically show higher Yule’s K values, indicating greater lexical repetition and lower linguistic variation, while real headlines exhibit marginally higher mean token and character counts, suggesting more elaborated expressions. Moreover, structural cues such as punctuation count, word-length variability, and the binary quote flag also contribute meaningfully to class separation. The presence of quotation marks and higher punctuation density tend to correlate with authentic reporting, where attribution and syntactic completeness are common. In contrast, deceptive headlines favor brevity, fewer unique words, and simpler structures—features that facilitate emotional appeal and faster reader engagement. These consistent trends across diverse sources underscore the generalizability of linguistic and stylistic indicators in detecting fake news in Arabic. (Fig 8) illustrates the Arabic Fake-News Datasets — Preprocessing Pipeline Summary. It demonstrates how all datasets were subjected to a unified, Arabic-aware text-cleaning process designed to preserve stylistic and linguistic cues essential for fake-news detection. The pie chart figure is weighted by dataset size, and each section highlights the dataset-specific preprocessing variations, showing that while all corpora follow the same normalization and cleaning principles, minor adjustments were made according to their content type and source domain.

### 3.6 Implementation details and computational environment

All experiments were implemented in Python using Google Colab as the primary execution environment. Classical and hybrid models were built with scikit-learn, while transformer models were implemented with the Hugging Face transformer library and trained with PyTorch. Feature ranking for engineered cues was performed using mutual information on training split only, and the selected top-*K* features were used consistently in hybrid and late-fusion settings. For transformer fine-tuning, GPU resources available through Google Colab were used to accelerate training and inference, while general preprocessing, feature extraction, and classical model training were also compatible with standard CPU execution (e.g., Intel Core i5-class hardware). All transformer fine-tuning experiments use a fixed random seed of 42(numpy, torch, and transformers are all seeded identically); for the unified corpus ablation study, results are additionally averaged over seeds {42, 52, 62} as described in the ablation study Section. For individual dataset evaluations (ANS, WELFake+LIAR, VERA–ARAB, AFND), the reported result is the single best run selected by development-set macro–F1 over the learning rate grid $\left\{10^{-5},2\times 10^{-5}\right\}$. The fine-tuning hardware was an NVIDIA T4 GPU (16 GB) via Google Colab; approximate wall-clock time per run was 20–25 minutes for smaller corpora (ANS) and 3–4 hours for the unified corpus. Moreover, all code and preprocessing scripts are publicly available in the project repository at https://github.com/AlbtoushEma2026/ArabicNlpCode, with the full pipeline notebook accessible directly. The Arabic-translated LIAR and WELFake datasets are released as a community contribution and are available for download.

## 4. Results

This section presents the experimental results for all datasets and discusses the main linguistic and modeling patterns observed during the experiments. We compare classical TF–IDF baselines, hybrid models that combine text representations with engineered indicators, and transformer-based models with and without feature fusion. We anchor the classical baseline on TF–IDF rather than static word embeddings (word2vec, GloVe, fastText): TF–IDF is a strong, transparent, and reproducible baseline that directly exposes the lexical and character-level regularities we analyse, whereas static embeddings assign a single context-independent vector per word and are poorly suited to short, morphologically rich, and code-switched Arabic headlines. Moreover, the contextual subword transformers evaluated here (MARBERT and AraELECTRA) already subsume the representational role of static embeddings while additionally modelling context; a direct comparison against static-embedding classifiers is left as future work. The presentation is organized by dataset in order to show how differences in domain, headline style, dialect variation, and numeric or temporal grounding influence detection performance and the relative effectiveness of each modeling approach. To ensure comparability across corpora, we follow a unified validation policy throughout the study. For the main experiments, models are selected on the validation split and reported on the held-out test split under the same train/validation/test protocol across datasets. The selected linear, hybrid, and transformer baselines were motivated by our previous headline-focused Arabic fake-news study [12], where these model families showed the strongest and most consistent performance, particularly on AFND, which remains the largest native Arabic headline dataset considered in this line of work. For the smaller ANS corpus, we additionally report stratified 5-fold cross-validation on the pooled Train+Dev portion as a supplementary robustness check, while preserving the official test split for final evaluation.

### 4.1 ANS dataset

For TF–IDF models, we encode ANS claims with a union of word 1–2 grams and character 2–5 grams to capture lexical and subword patterns in short claims. For the Hybrid models, we concatenate the strongest engineered cues identified for ANS (numeric density and temporal markers, negation/modality usage, and compact length statistics), emphasizing structural signals over social or dramatic punctuation. Each classifier (Logistic Regression or LinearSVC) is tuned over *C* via validation macro–F1, then refit on Train+Val and evaluated once on the held-out Test split. The ANS corpus exhibits a 1:2.08 class imbalance (fake: 1,474; real: 3,071), making accuracy an unreliable sole metric. We therefore report macro-averaged precision, recall, and F1 as primary metrics throughout, since macro-averaging weights both classes equally regardless of support size — directly penalizing models that neglect the minority fake class. Weighted F1 was not adopted as it would inflate scores in favour of the majority real class and obscure poor minority-class recovery. Per-class F1 scores for fake and real classes are additionally reported in Table 12 to allow direct inspection of minority-class performance. AUC-ROC is reported for all models that produce calibrated probability scores; LinearSVC does not natively output calibrated probabilities and is marked N/A. For linear classifiers, class imbalance is compensated via class_weight = “balanced” in both Logistic Regression and LinearSVC. Transformer models are fine-tuned with an inverse-frequency weighted cross-entropy loss, making explicit oversampling unnecessary and consistent with standard practice for moderately imbalanced corpora under deep learning fine-tuning [40]. The resulting test metrics appear in Table 12. Among linear baselines, TF–IDF models achieve the highest accuracy (0.6725) and macro–F1 (≈0.638) within this group, with Hybrid variants showing slightly lower overall accuracy despite marginal gains in minority-class (fake) F1 recall. Notably, the per-class breakdown reveals that all linear and hybrid models struggle with the minority fake class (F1_fake_ ≈ 0.52–0.54), while achieving substantially higher F1 on the majority real class (≈0.73–0.75), confirming that accuracy alone would be misleading under this imbalance. Transformer models decisively outperform all linear baselines: AraELECTRA (text-only) achieves the best accuracy (0.7407) and AUC-ROC (0.7809), while AraELECTRA with top-10 feature fusion attains the best macro–F1 (0.7026), best F1_fake_ (0.5993), and best AUC (0.7874), confirming that subword representations substantially improve minority-class recovery. MARBERT performs consistently lower than AraELECTRA on this dataset.

**Table 12:** Held-out test performance on ANS.

Models	Best *C*	Acc.	P_mac_	R_mac_	F1_fake_	F1_real_	F1_mac_	AUC
TF–IDF + LR	0.5	0.6725	0.6360	0.6422	0.5270	0.7496	0.6383	0.6682
TF–IDF + LinearSVC	0.0625	0.6725	0.6351	0.6405	0.5240	0.7504	0.6372	N/A
Hybrid + LR	1	0.6571	0.6338	0.6477	0.5439	0.7254	0.6346	0.7090
Hybrid + LinearSVC	0.125	0.6615	0.6350	0.6476	0.5417	0.7317	0.6367	N/A
AraELECTRA (text)	—	**0.7407**	**0.7056**	0.6981	0.5931	0.8097	0.7014	0.7809
MARBERT (text)	—	0.6967	0.6685	0.6823	0.5818	0.7621	0.6719	0.7477
AraELECTRA (top-10)	—	0.7385	0.7037	**0.7016**	**0.5993**	**0.8059**	**0.7026**	**0.7874**
MARBERT (top-10)	—	0.7209	0.6865	0.6919	0.5890	0.7887	0.6888	0.7617

Linear and hybrid models use TF–IDF with word unigrams/bigrams and character n-grams; hybrid variants additionally incorporate the top-*K* numeric and stylistic cues. All metrics are macro-averaged over both classes. Per-class F1 scores are reported to diagnose minority-class recovery under the 1:2.08 class imbalance. P_mac_: macro precision; R_mac_: macro recall; F1_fake_/F1_real_: per-class F1; N/A: LinearSVC does not produce calibrated probabilities.

For the ANS dataset, we supplemented the original held-out evaluation with stratified 5-fold cross-validation on the pooled Train+Dev split, while keeping the official Test split unchanged for final assessment. This preserves the unified validation strategy used across datasets and provides an additional robustness check for the smaller corpus. The cross-validation results support the same overall conclusion: transformer models remain clearly stronger than the linear and hybrid baselines, with AraELECTRA achieving the best performance (CV macro-F1 =0.6375±0.0162, Test macro-F1 = 0.7014), followed by MARBERT (CV macro-F1 =0.6269±0.0090, Test macro-F1 = 0.6719). By comparison, the linear and hybrid models remain substantially lower.

### 4.2 Welfake and liar datasets

We extract entity, punctuation, and lexical features tailored to short Arabic headlines: counts/flags for URLs, mentions, hashtags, percentages, money symbols, dates/times; punctuation cues (question/exclamation marks, ellipses, multi–punctuation runs); quotation usage; English-token presence and ALL-CAPS forms; numeric cues (numbers and four-digit years); as well as negations, modals, hedges, and clickbait triggers (e.g., “عاجل”, “لن تصدق”). Overall descriptive statistics (over all classes) show low but informative incidence of these cues. Critically, per-class means reveal clear stylistic asymmetries that are characteristic of fake headlines:

- **Amplification and sensationalism:** fake > real for question marks (0.0536 vs. 0.0199), exclamation marks (0.0431 vs. 0.0019), ellipsis (0.0643 vs. 0.0032), multi-punctuation runs (0.0648 vs. 0.0034), and hashtags (0.0067 vs. 0.0002).
- **Quotation framing:** fake uses quotes more heavily (mean n_quotes=0.4794 vs. 0.2343; has_quotes=0.1976 vs. 0.1024), consistent with attributed claims and sensational framing.
- **Cross-lingual/MT artifacts and quantification:** fake shows more English tokens (0.2523 vs. 0.1194) and higher numeric density (n_numbers=0.2510 vs. 0.1684); years are slightly more common in fake (0.0305 vs. 0.0229).
- **Length effects:** fake headlines are longer in characters and tokens (clean tokens 12.86 vs. 11.49), while average token length is slightly shorter (4.96 vs. 5.08), aligning with teaser-like constructions.
- **Factuality cues:** real headlines are marginally higher in temporal markers (dates/times) and hedging terms.

These patterns indicate that deceptive headlines rely more on emphatic and dramatic stylistic devices, while real headlines favor concise, temporally grounded phrasing. Such cues are vital for short-text classification where syntactic depth is limited. In subsequent sections we will report model performance (TF–IDF + LR/SVM; MARBERT; AraBERTv2) that exploits these features; here, our focus is to document the translation process, the resulting corpus characteristics, and the distributional behavior of features that are most indicative of deception in Arabic headlines. Table 13 shows Held–out test performance on WELFake+LIAR (Helsinki-MT, stratified 80/10/10).

**Table 13:** Held-out test performance on WELFake+LIAR (Helsinki-MT, stratified 80/10/10).

Model	Features	Params	Accuracy	Precision (macro)	Recall (macro)	F1 (macro)
TF–IDF + LR	TF–IDF (text only)	C = 1	0.8141	0.8135	0.8151	0.8137
TF–IDF + LinearSVC	TF–IDF (text only)	C = 0.125	0.8163	0.8157	0.8173	0.8159
Hybrid + LR	TF–IDF + Top–10 cues	C = 1, K = 10	0.8190	0.8182	0.8197	0.8185
Hybrid + LinearSVC	TF–IDF + Top–10 cues	C = 0.0625, K = 10	0.8155	0.8151	0.8168	0.8151
AraELECTRA (text)	Subword (WordPiece)	epochs=4, lr=$2\;\times \;10^{-5}$, max_len=192	0.8550	0.8551	0.8545	0.8547
MARBERT (text)	Subword (WordPiece)	epochs=4, lr=$2\;\times \;10^{-5}$, max_len=192	0.8500	0.8498	0.8500	0.8499
AraELECTRA (top–10)	Subword + Top–10 features	epochs=4, lr=$2\;\times \;10^{-5}$, max_len=192, K=10	0.8562	0.8560	0.8560	0.8560
MARBERT (top–10)	Subword + Top–10 features	epochs=4, lr=$2\;\times \;10^{-5}$, max_len=192, K=10	0.8541	0.8539	0.8540	0.8539

TF–IDF uses word 1–2 grams + character 3–6 grams.

### 4.3 VERA–ARAB dataset

We process VERA–ARAB with an Arabic aware normalization pipeline mirroring our code for other corpora: we replace surface artifacts with placeholders (<URL > , < EMAIL > , user mentions, hashtags; also collapse percents, money, dates, and times), unify Arabic digits to Latin, remove diacritics and tatwīl, normalize letter variants, collapse elongations, and tidy whitespace. We then drop empty, duplicate, and ultra short texts to preserve alignment between cleaned text and engineered features. From the cleaned tweet/headline texts, we extract a compact suite of lexical–stylistic cues including length and diversity (character/word counts, Guiraud’s R, TTR), character composition (Arabic/Latin/digit/punctuation shares), discourse markers (URLs, hashtags, mentions, dates/times, money, percent), orthographic signals (elongations, quotes, question/exclamation), and lexicon hits (negation, modality, reporting, sensationalism). For fusion models, we select the top *K* numeric features on the train split (default *K* = 10), Table 14 summarizes held out performance.

**Table 14:** Held-out test performance on VERA_ARAB.

Models	Features	Params	Accuracy	Precision (macro)	Recall (macro)	F1 (macro)
TF–IDF + LogisticRegression	word(1–2) + char_wb(3–6)	C = 1.0, L2, liblinear, class_wt = balanced, max_iter = 2000	0.7356	0.7361	0.736838	0.7355
TF–IDF + LinearSVC	word(1–2) + char_wb(3–6)	C = 1.0, class_wt = balanced, max_iter = 5000	0.7488	0.7490	0.7498	0.7487
Hybrid (TF–IDF + Top-10) + LogisticRegression	TF–IDF + Top-10 cues	C = 1.0, L2, liblinear, class_wt = balanced, max_iter = 2000	0.7533	0.7531	0.7540	0.7530
**Hybrid (TF–IDF + Top-10) + LinearSVC**	**TF–IDF + Top-10 cues**	**C = 1.0, class_wt = balanced, max_iter = 5000**	**0.7621**	**0.7613**	**0.7613**	**0.7613**
AraELECTRA (text)	subword (WordPiece)	epochs=4, lr$=2\;\times \;10^{-5}$, max_len=192	0.7268	0.7335	0.7310	0.7266
MARBERT (text)	subword (WordPiece)	epochs=4, lr$=2\;\times \;10^{-5}$, max_len=192	0.7356	0.7361	0.7368	0.7355
AraELECTRA (top-10)	subword + Top-10 features	epochs=4, lr$=2\;\times \;10^{-5}$, max_len=192, *K*=10	0.7136	0.7132	0.7109	0.7114
**MARBERT (top-10)**	**subword + Top-10 features**	**epochs=4, lr$=2\;\times \;10^{-5}$, max_len=192, *K*=10**	**0.7841**	**0.7867**	**0.7841**	**0.7841**

Hybrid rows fuse TF–IDF with the top-*K* numeric/stylistic cues (Top-10). Transformer rows report fine-tuned models with “top-10” fusion of the top engineered features.

Among linear baselines, TF–IDF with character n grams (3–6, within word boundaries) and word unigrams/bigrams reaches Acc/F1 ≈ 0.749 with LinearSVC, and improves further with late fusion of the Top 10 numeric cues (Acc/F1 = 0.762). Transformer models are stronger overall: MARBERT (text only) attains Acc/F1 ≈ 0.736, while MARBERT with Top 10 late fusion achieves the best single model score (Acc/F1 = 0.784). AraELECTRA trails both MARBERT variants, and its fusion gains are smaller. Here, late fusion refers to the strategy of first obtaining a probability score from the transformer backbone independently, then concatenating the top 10 engineered feature vector to the transformer’s final representation before the classification layer, allowing stylistic and semantic signals to complement each other without modifying the transformer architecture itself.

These improvements are consistent with the class wise trends in our feature analysis (Table 9): Fake texts are typically shorter and lexically poorer (fewer unique words, lower token counts, lower Guiraud’s R), show greater redundancy (higher compression ratio), and differ in character composition (higher Latin%). Real texts exhibit richer lexical variety and distinct punctuation/emoji usage. Such low dimensional, orthogonal cues complement the subword semantics captured by MARBERT, explaining the strong lift from late fusion. In contrast, TF–IDF already encodes many surface regularities, so its hybrid gains, while consistent, are more modest. Overall, MARBERT + Top 10 engineered features (late fusion) is the preferred model for VERA–ARAB, with TF–IDF + Top 10 + LinearSVC serving as a competitive, lightweight alternative when resources are limited.

### 4.4 AFND dataset

On the AFND headlines, short text alone carries clear veracity signal. Classical TF–IDF baselines are competitive (≈0.70–0.72 accuracy), with a small lift from fusing the Top-10 engineered cues. Transformer models outperform them: AraELECTRA and MARBERT reach ≈0.74–0.75 accuracy, with text-only runs slightly edging their feature-fusion variants. The most discriminative cues reflect style and composition rather than content—Fake tends to have a marginally higher share of Latin characters, more punctuation and numbers, and slightly shorter character sequences; Real shows a bit richer vocabulary (higher unique words and token count) and lower compression. Overall, headline-only detection on AFND benefits from strong subword encoders, while compact stylistic features add complementary—but modest—gains. Table 15 shows the Held-out test performance on AFND dataset.

**Table 15:** Held-out test performance on AFND (stratified 80/10/10). TF–IDF uses word 1–2 grams + character char_wb 3–6 grams.

Model	Features	Params	Accuracy	Precision (macro)	Recall (macro)	F1 (macro)
TF–IDF + LR	TF–IDF (text only)	C = 1	0.7173	0.7138	0.7143	0.7141
TF–IDF + LinearSVC	TF–IDF (text only)	C = 1	0.7009	0.6971	0.6972	0.6972
Hybrid + LR	TF–IDF + Top–10 cues	C = 1, K = 10	0.7181	0.7146	0.7149	0.7147
Hybrid + LinearSVC	TF–IDF + Top–10 cues	C = 1, K = 10	0.7008	0.6970	0.6972	0.6971
AraELECTRA (text)	Subword (WordPiece)	epochs=4, lr=$2\;\times \;10^{-5}$, max_len=192	0.7495	0.7547	0.7362	0.7389
AraELECTRA (top–10)	Subword + Top–10 features	epochs=4, lr=$2\;\times \;10^{-5}$, max_len=192, K=10	0.7486	0.7464	0.7417	0.7432
MARBERT (top–10)	Subword + Top–10 features	epochs=4, lr=$2\;\times \;10^{-5}$, max_len=192, K=10	0.7470	0.7438	0.7429	0.7433
MARBERT (text)	Subword (WordPiece)	epochs=4, lr=$2\;\times \;10^{-5}$, max_len=192	0.7442	0.7413	0.7385	0.7396

### 4.5 Unified corpus (ANS, AFND, LIAR, WELFake, and VERA-ARAB) datasets

Building on this corpus, we evaluated classical TF–IDF baselines and subword transformers; the results in Table 16 show a clear advantage for the latter. While TF–IDF with Logistic Regression or LinearSVC yields solid and very consistent performance (Accuracy ≈0.725–0.731, macro-F1 ≈0.723–0.729), both AraELECTRA and MARBERT improve markedly, reaching ~0.768–0.770 accuracy and macro-F1 ~0.764–0.767 on the unified split. The best overall result is obtained by MARBERT (text-only), with 0.7695 accuracy and a balanced precision/recall profile, indicating that large Arabic pretraining captures broad stylistic and lexical cues present across sources. Adding the Top–10 engineered features to the transformers produces only marginal fluctuations on the unified corpus (ΔF1 ≤ 0.002 for both MARBERT and AraELECTRA). Crucially, McNemar’s exact test confirms that these differences are not statistically significant, indicating that subword encoders already internalize the surface-level stylistic cues captured by the engineered features. This finding is consistent across both old and new feature sets, establishing the robustness of the conclusion.. Taken together, Table 16 supports two conclusions: (i) unifying heterogeneous sources improves robustness enough for pretrained Arabic transformers to generalize well, and (ii) classical feature engineering remains competitive and interpretable at lower computational cost, but modern transformers extract richer signals from the same lightly normalized text.

**Table 16:** Held-out test performance on the unified corpus (ANS, AFND, LIAR, WELFake and VERA-ARAB).

Model	Features	Params	Accuracy	Precision (macro)	Recall (macro)	F1 (macro)
TF–IDF + LR	TF–IDF (text only)	C = 1	0.7301	0.7285	0.7285	0.7285
TF–IDF + LinearSVC	TF–IDF (text only)	C = 1	0.7247	0.7231	0.7231	0.7231
Hybrid + LR	TF–IDF + Top–10 cues	C = 1, K = 10	0.7306	0.7290	0.7292	0.7291
Hybrid + LinearSVC	TF–IDF + Top–10 cues	C = 1, K = 10	0.7249	0.7233	0.7234	0.7234
AraELECTRA (text)	Subword (WordPiece)	epochs=4, lr=$2\;\times \;10^{-5}$, max_len=192	0.7684	0.7706	0.7629	0.7644
AraELECTRA (top–10)	Subword + Top–10 features	epochs=4, lr=$2\;\times \;10^{-5}$, max_len=192, K=10	0.7694	0.7685	0.7666	0.7673
MARBERT (text)	Subword (WordPiece)	epochs=4, lr=$2\;\times \;10^{-5}$, max_len=192	0.7695	0.7703	0.7650	0.7663
MARBERT (top–10)	Subword + Top–10 features	epochs=4, lr=$2\;\times \;10^{-5}$, max_len=192, K=10	0.7680	0.7671	0.7654	0.7660

### 4.6 Ablation study: Feature fusion and statistical significance

Feature fusion is motivated by the hypothesis that compact stylistic cues—lexical repetition, punctuation density, and sentence structure—carry signal orthogonal to subword semantics, providing complementary discriminative information. We expected modest gains for linear models, since TF-IDF does not directly encode these cues, and negligible gains for transformers, since MARBERT and AraELECTRA already implicitly encode surface-level patterns through self-attention. To evaluate this, the official test split (*n* = 44,922) was held out throughout. For linear and hybrid models, we pooled the Train and Dev portions and applied stratified 5-fold cross-validation, varying the fused feature count K∈{5,10,15,20} and selecting the best *K* by mean macro-F1 before a single final evaluation on the held-out test set. For transformer models, all runs used three random seeds (42, 52, 62) and results are averaged over those seeds. Every pairwise comparison is assessed with McNemar’s exact test (two-sided binomial). Table 17 summarises the full ablation on the unified corpus. Three clear findings emerge. *First*, transformers decisively outperform linear baselines: the best linear model (Hybrid+LR, F1 = 0.733) versus the best transformer (MARBERT text-only, F1 = 0.770) yields McNemar p≪0.001, confirming the gap is statistically reliable and not attributable to sampling variation. *Second*, feature fusion does not significantly improve transformers on the unified corpus. McNemar’s test shows no significant difference between text-only and top-10 fusion for either backbone: AraELECTRA (*b* = 1447, *c* = 1429, *p* = 0.751) and MARBERT (*b* = 1608, *c* = 1602, *p* = 0.930). This result held in a supplementary sensitivity check using a richer 36-feature alternative set, confirming the finding is robust to feature engineering choices (AraELECTRA *p* = 0.491; MARBERT *p* = 0.627). *Third*, the backbone choice is itself a statistically significant factor: AraELECTRA versus MARBERT (majority vote over three seeds) yields $p=8.4\times 10^{-9}$, establishing MARBERT as the significantly stronger backbone for this corpus. Taken together, feature fusion is most valuable when the dataset exposes cues orthogonal to subword semantics—most clearly in VERA-ARAB, where MARBERT+top-10 reaches 0.7841 accuracy. On the large, heterogeneous unified corpus, however, transformers already encode the relevant surface signals, and adding handcrafted features provides no statistically reliable benefit. This finding indicates that practitioners targeting multi-source Arabic headline corpora should invest in backbone selection rather than feature engineering.

**Table 17:** Ablation study on the unified corpus (ANS + AFND + LIAR + WELFake + VERA-ARAB).

Model	Features	Params	Accuracy	Precision (macro)	Recall (macro)	F1 (macro)	McNemar vs text-only
*Linear baselines*							
TF-IDF + LR	TF-IDF (text only)	*C* = 1	0.735	0.733	0.733	0.733	—
TF-IDF + LinearSVC	TF-IDF (text only)	*C* = 1	0.724	0.723	0.723	0.723	$p=5.6\times 10^{-11}$ †
*Hybrid baselines (TF-IDF + top-10 features)*							
Hybrid + LR	TF-IDF + Top-10	*C* = 1	0.735	0.734	0.733	0.733	*p* = 0.593 *ns*
Hybrid + LinearSVC	TF-IDF + Top-10	*C* = 1	0.726	0.724	0.724	0.724	*p* = 0.017 †
*Transformer models — mean over 3 seeds; epochs = 4, lr$=2\times 10^{-5}$, max_len = 192*							
AraELECTRA (text)	Subword (WordPiece)	—	0.764	0.767	0.758	0.760	—
AraELECTRA (top-10)	Subword + Top-10	*K* = 10	0.762	0.764	0.757	0.758	*p* = 0.751 *ns*
MARBERT (text)	Subword (WordPiece)	—	0.775	0.780	0.768	0.770	—
MARBERT (top-10)	Subword + Top-10	*K* = 10	0.773	0.774	0.769	0.771	*p* = 0.930 *ns*

Transformer results are averaged over three seeds (42, 52, 62). The McNemar column reports exact two-sided binomial *p*-values comparing each fusion variant against its text-only counterpart (*n* = 44,922); “—” indicates the text-only reference row for that backbone. † statistically significant (*p* < 0.05); *ns* not significant. Backbone comparison (AraELECTRA vs. MARBERT majority vote): $p=8.4\times 10^{-9}$ (†). Best linear vs. best transformer (Hybrid+LR vs. MARBERT text-only): p≪0.001 (†).

## 5. Discussion

This study investigates the detection of fake news headlines only in four Arabic datasets of distinct style and origin: ANS, WELFake + LIAR (machine translated), VERA-ARAB, and AFND – and a unified corpus that combines them under a consistent processing pipeline. We evaluate classical linear models using TF–IDF, hybrid approaches that incorporate a compact set of engineered numeric and stylistic features, and transformer baselines with and without feature fusion. As visualized in Fig 10, length- and numeric-based features form a stable core across all corpora, while punctuation and social markers co-vary more strongly in fake headlines. These correlations reinforce the idea that deception in headlines is expressed less through lexical novelty and more through stylistic emphasis, density, and information packaging. Three high-level findings emerge from the results. First, the cues that most reliably distinguish fake from real headlines are compact, surface-level features rather than topical content. Character length and token count are strong indicators, with fake headlines typically longer yet composed of shorter words. Lexical repetitiveness, measured by Yule’s *K*, consistently increases in fake content, reflecting redundant word choices and exaggerated phrasing. Punctuation-based cues are among the most striking markers of deceptive tone: as shown in Table 8, exclamation marks appear **22.7 times** more frequently in fake headlines than in real ones (0.0431 vs. 0.0019), ellipsis **20.1 times** more frequently (0.0643 vs. 0.0032), and multi-punctuation runs **19.1 times** more frequently (0.0648 vs. 0.0034). These extraordinary ratios underscore that deceptive framing relies heavily on emphatic and dramatic punctuation devices to manufacture urgency and emotional engagement. In contrast, real headlines tend to have steadier proportions of Arabic letters, fewer punctuation bursts, and more factual numeric or temporal anchors (numbers, years, and dates). Beyond punctuation, logical and modal markers further differentiate the two classes: as shown in Table 6, real headlines in the ANS corpus exhibit significantly higher negation counts (0.993 vs. 0.881), more modal verb usage (0.044 vs. 0.035), and more hedging terms (0.009 vs. 0.005), reflecting greater epistemic commitment and factual qualification — linguistic behaviors consistent with verified reporting rather than speculative or sensational framing. These findings align with prior psycholinguistic work showing that deceptive writing tends to favor expressive density over factual conciseness. Second, model behavior follows a stable hierarchy: transformer models outperform TF–IDF baselines, and hybrid TF–IDF models show only marginal gains. On the **ANS** dataset, linear baselines achieve about 0.67 accuracy, while transformers perform substantially better (AraELECTRA 0.7407, MARBERT 0.6967). Adding the top-10 engineered features produces a marginal decline for AraELECTRA, indicating that stylistic cues are already partially encoded in subword representations. On **WELFake+LIAR**, TF–IDF is strong (0.815–0.819) and the transformers reach 0.855 accuracy, but the fusion effects are negligible (≤0.004). Notably, the translated corpus also reveals a cross-lingual stylistic asymmetry: as reported in Table 8, fake headlines contain **twice as many residual English tokens** as real headlines (0.252 vs. 0.119), a pattern that reflects not only MT artifacts but also genuine tendencies in deceptive headlines to incorporate foreign-language terms, brand names, and untranslated ALL-CAPS expressions as attention-grabbing devices. The clearest benefit appears in **VERA–ARAB**, where numeric and temporal cues are more salient: LinearSVC rises from 0.7488 to **0.7621**, and **MARBERT+top-10** reaches the overall best score of **0.7841**. In contrast, AraELECTRA+top-10 declines on VERA–ARAB (0.7136 vs. 0.7268 for text-only), indicating that late fusion is not equally effective. This decline is unlikely to be explained by poor feature quality alone, since the same top-10 cues improve both the linear baseline and MARBERT on the same VERA–ARAB dataset. A more plausible explanation is a backbone-specific mismatch between the injected lexical–stylistic features and AraELECTRA’s learned representation under the multi-dialect and social-media conditions of VERA–ARAB. This dataset is the most socially noisy and dialect-heavy corpus in the paper, and its most discriminative cues are highly sensitive to register, including hashtags, URLs, emoji usage, Latin-script proportion, punctuation, and compression-related signals. In particular, Table 9 reveals a culturally distinctive pattern: real headlines in VERA–ARAB contain **46% more emojis** than fake ones (0.570 vs. 0.391), suggesting that authentic Arabic social-media reporting employs emojis as communicative and expressive markers, whereas fake content relies more on hashtags (1.187 vs. 0.980) and Latin-script artifacts to attract attention. These cues appear to complement MARBERT, but interact less effectively with AraELECTRA. Some overfitting to sparse social-media markers may also play a role; however, the overall pattern is more consistent with fusion sensitivity under domain and register shift than with simple feature collinearity alone. For **AFND**, where social signals (hashtags, mentions, URLs) dominate but are weakly correlated with class, all model families cluster closely (TF–IDF ≈0.70–0.72; transformers ≈0.74–0.75) with negligible improvement from feature fusion. The **unified corpus** confirms these tendencies: subword transformers converge near 0.768–0.770 accuracy with minimal (≤0.002) movement from fusion, while TF–IDF and hybrid variants remain around 0.724–0.731. Third, feature fusion proves most beneficial when the dataset foregrounds verifiable numeric or temporal anchors and when the transformer backbone can leverage low-dimensional signals without over-regularization. This explains the consistent improvement of VERA–ARAB under MARBERT + top–10 and the small gain of AraELECTRA on ANS. In stylistically homogeneous datasets such as WELFake+LIAR or socially noisy ones like AFND, explicit fusion adds little because the stylistic variance is either already captured by token-level statistics or uninformative for classification.

**Fig 9:**
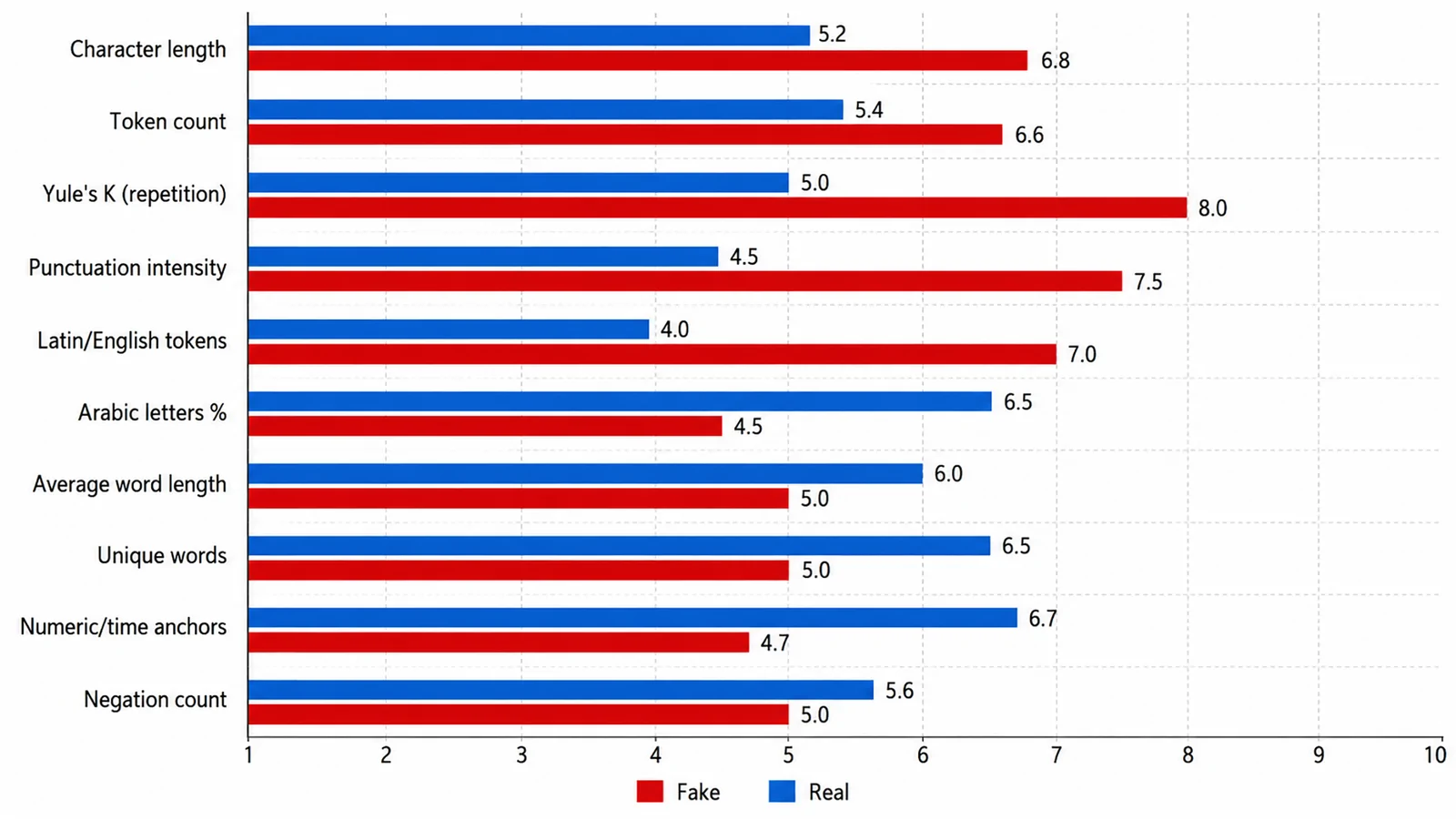
Linguistic and stylistic indicators of fake headlines in Arabic news.

Across corpora, the strongest and most portable discriminators are **character length**, **token count**, **Yule’s *K***, **punctuation count** and **exclamation flag**, **Latin/Arabic character proportions**, and **negation count**. Dataset-specific reinforcement occurs for **numbers**, **four-digit years**, and **date/time mentions**. These cues are compact (the top-10 suffices), interpretable, and robust under normalization. They complement transformer embeddings by providing interpretable structure that captures intensity, redundancy, and factual grounding—dimensions that pure subword models treat implicitly. In practice, effective headline-only pipelines should (i) build strong TF–IDF baselines using word and character *n*-grams, (ii) fine-tune a transformer backbone with balanced sequence lengths, and (iii) optionally append a small curated feature set when the dataset shows strong numeric or stylistic contrast. Relying on rare, dataset-specific artifacts (hashtags, mentions, URLs) offers little value. Overall, modern subword models internalize most stylistic variation, but explicit fusion remains useful when factual and emotional cues co-occur in short texts. The detection of fake news headlines in Arabic benefits from integrating lexical, stylistic, and structural signals. Transformers deliver the highest accuracy overall, while feature fusion yields targeted gains on corpora with prominent numeric and temporal patterns (VERA–ARAB; MARBERT+top-10 at **0.7841**), moderate improvement for formal claim-based datasets (ANS; AraELECTRA+top-10 at **0.7473**), and minimal change elsewhere. (Fig 9) illustrates Linguistic and stylistic indicators distinguishing fake and real Arabic news headlines on a 1–10 normalized scale. Each pair of bars represents the relative prominence of a given feature across all datasets, comparing fake (red) and real (blue) headlines. Features such as Yule’s K, punctuation intensity, and Latin/English token share are consistently higher in fake headlines, reflecting redundancy and expressive tone, whereas real headlines exhibit greater Arabic letter proportion, numeric and temporal anchors, and lexical diversity.f

**Fig 10:**
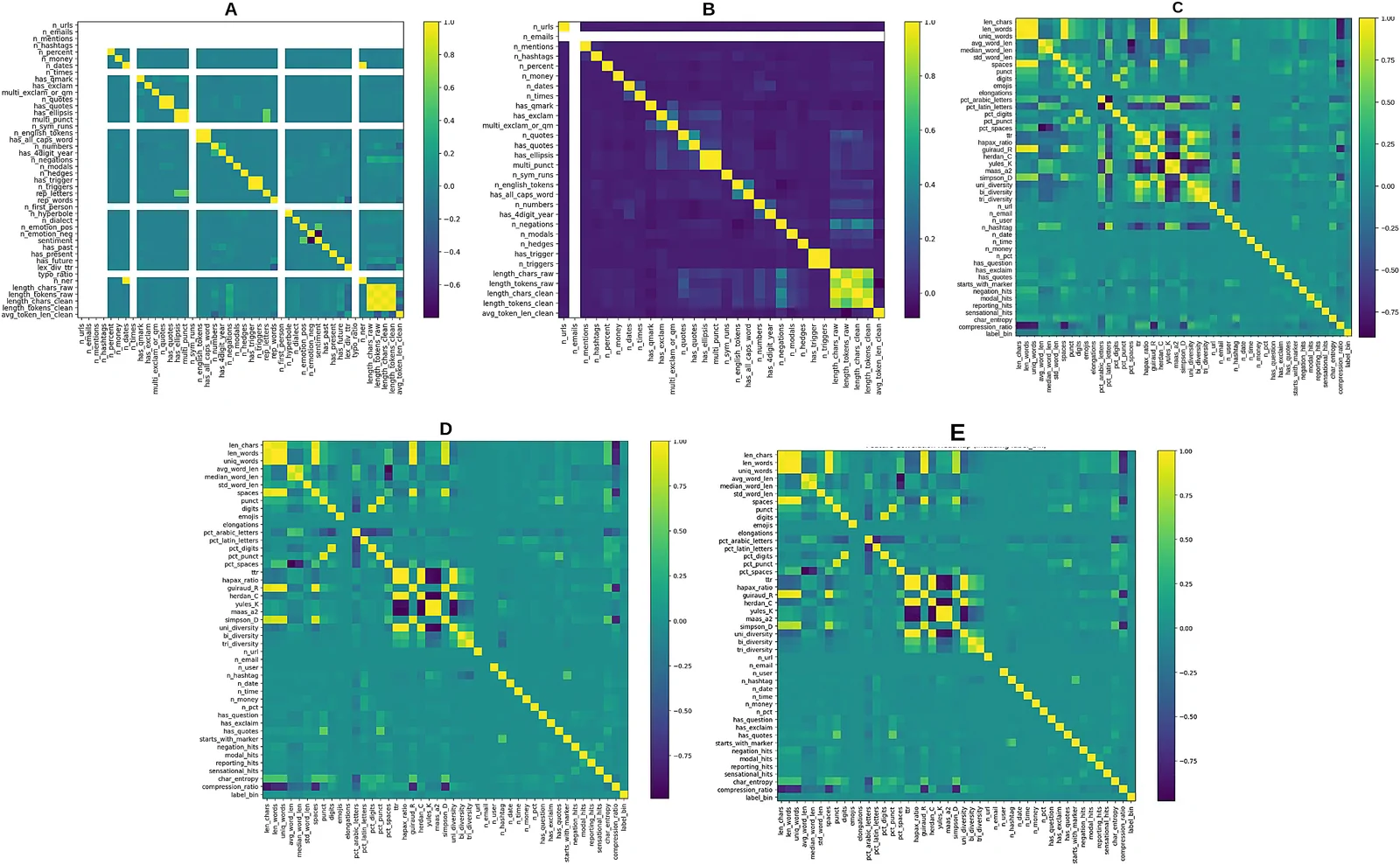
Correlation structures across datasets: (A) ANS; (B) LIAR+WELFake; (C) VERA–ARAB; (D) AFND; (E) unified all-dataset view (ANS, LIAR+WELFake, VERA–ARAB, and AFND).

The visualization highlights interpretable, cross corpus cues that differentiate deceptive from factual content. The consistent stability of length and numeric features across datasets, together with the stylistic co-movement seen in Fig 10 demonstrates that fake headlines can be effectively detected by coupling subword semantics with a small, interpretable set of linguistic cues that generalize across domains.

### 5.1 Comparison with previous studies

As summarized in Table 18, most prior studies on Arabic fake news detection have concentrated on *full news articles* rather than headlines. To the best of our knowledge, no previous research has exclusively addressed the detection of fake news using *Arabic headlines* alone. Existing works, such as [31], primarily relied on headline–article pairs within restricted and domain-specific datasets, while others focused on single-topic or small-scale corpora. We note that the number of directly comparable baselines is limited by the novelty of the task: because headline-only Arabic fake-news detection has not previously been addressed in isolation, only two prior studies permit a like-for-like comparison ([31], headline–article pairs, 2022; and [12], headline-only, 2025). Other recent systems target full-article, multimodal, or multi-source settings on different corpora and label schemes, which makes a direct numeric comparison methodologically unsound; we therefore position them qualitatively rather than as controlled baselines, and situate our results within the most recent literature in Table 3. For instance, [31] evaluated hybrid CNN–LSTM architectures on a limited Syrian war dataset, achieving around 70% accuracy when both headlines and articles were combined. Similarly, [12]—our earlier study—investigated multiple architectures including SVM, CNN, LSTM, and Arabic transformer models such as AraBERTv2 and AraELECTRA using headline-only inputs from the AFND dataset, achieving up to 70.4% accuracy with AraBERTv2 and 70.24% with MARBERTv2. These results highlighted the feasibility of headline-based classification but also revealed performance constraints caused by the short and context-limited nature of headlines. In the current work, we extend our previous experiments to incorporate a richer set of linguistic and stylistic indicators, specifically the **top-10 engineered features** most correlated with deception cues. By integrating these interpretable linguistic features with subword-level transformer embeddings, we observe measurable improvements in performance. Our best-performing models, **AraELECTRA** and **MARBERT**, achieved accuracies of 0.7694 and 0.7680 respectively—surpassing earlier single-dataset headline baselines. Unlike prior research that depended on single-domain or monolingual sources, our unified approach aggregates multiple corpora (AFND, ANS, LIAR-Arabic, and WELFake-Arabic), spanning diverse topics, dialects, and writing styles. This heterogeneity enhances model robustness and improves the generalization capability of transformer-based architectures. The results reinforce the dominance of transformer models in capturing subtle linguistic and stylistic nuances in short Arabic texts, demonstrating their advantage over traditional machine and deep learning baselines. The integration of top-ranked linguistic features with transformer embeddings leads to targeted performance gains in specific corpus conditions. It represents a step toward scalable and domain-agnostic fake news detection in Arabic.

**Table 18:** Arabic fake news detection studies (2020–2025) focusing on headline or headline–article inputs, showing models, linguistic features, and accuracy results.

Reference	Input Type	Model(s)	Linguistic Features	Results (Acc)
[31]	Headline + Article	LSTM, CNN, CNN–LSTM	Sequence-based deep models (no handcrafted features)	70% (CNN–LSTM)
[12]	Headline Only	SVM, CNN, LSTM, AraBERTv2, AraELECTRA	Linguistic	70.4% (AraBERTv2), 70.24% (MARABERTV2), LR WITH TF-IDF 67.27% all on AFND dataset
**Our Model**	Headline Only	**AraELECTRA**, **MARBERT**	Linguistic, stylistic cues	LR With TF-IDF: 0.7306, AraELECTRA: 0.7694, MARBERT: 0.7680

## 6. Conclusion

Across all five headline corpora—ANS, AFND, LIAR+WELFake (machine–translated), VERA–ARAB, and their unified combination—our findings converge on a consistent pattern of performance and linguistic behavior. Transformer backbones such as AraELECTRA and MARBERT decisively outperform classical TF–IDF baselines, achieving accuracies around **0.768–0.770** on the unified corpus compared to **0.724–0.731** for linear TF–IDF models. This performance gap confirms that subword representations effectively capture both semantic and stylistic regularities even in short, title‐level texts. Compact engineered features—covering length and density (character and token counts), lexical repetition (Yule’s *K*), punctuation emphasis, and numeric/temporal anchors—reveal stable Fake–Real asymmetries across all datasets. Fake headlines tend to be slightly longer yet composed of shorter words, exhibit higher lexical redundancy, and use more punctuation and Latin characters. Real headlines are more lexically balanced and rely more on factual numeric expressions and steady Arabic‐letter composition. These consistent stylistic tendencies are further reflected in the correlation structures visualized in (Fig 10). where length and numeric cues form a stable core across corpora, while punctuation and social markers co-vary most strongly in fake content. While transformer baselines already internalize much of this stylistic information, fusion of the top-*K* engineered features provides targeted benefits. The effect is most visible in datasets rich in numeric or temporal anchors, particularly **VERA–ARAB** (MARBERT + top-10 → 0.7841 accuracy), and marginal in more homogeneous or socially noisy corpora such as AFND and the unified pipeline, where gains remain within ±0.002. Even when accuracy differences are small, the inclusion of interpretable features enhances explainability and facilitates monitoring for domain drift or emerging manipulation styles. In practical terms, the optimal configuration for headline-only Arabic misinformation detection is a transformer backbone—preferably MARBERT or AraELECTRA—supplemented by a lightweight, interpretable feature bundle capturing length, lexical density, punctuation, and numeric grounding. This combination balances **accuracy, efficiency, and transparency**, yielding a robust framework for large-scale detection of deceptive or sensational Arabic headlines. Future work may extend this framework to multimodal fake news detection, more robust cross-dialectal adaptation, and broader ablation studies that examine the contribution of individual feature groups and fusion settings in greater detail, further advancing low-resource Arabic NLP.
